# Two‐Stage Mixed‐Dye‐Based Isothermal Amplification with Ribonuclease‐Cleavable Enhanced Probes for Dual‐Visualization Detection of SARS‐CoV‐2 Variants of Interest

**DOI:** 10.1002/advs.202401988

**Published:** 2024-06-03

**Authors:** Xiong Ding, Yaru Wang, Yuxin Gui, Chuankun Yang

**Affiliations:** ^1^ Key Laboratory of Environmental Medicine and Engineering Ministry of Education Department of Nutrition and Food Hygiene School of Public Health, Southeast University Nanjing 210009 P. R. China; ^2^ Center of Clinical Laboratory Medicine Zhongda Hospital, Southeast University Nanjing 210009 P. R. China

**Keywords:** dual‐visualization detection, nucleic acid amplification, REP‐TMAP, ribonuclease‐cleavable enhanced probes, SARS‐CoV‐2 variants of interest

## Abstract

Rapid and visual detection of SARS‐CoV‐2 variants is vital for timely assessment of variant transmission in resource‐limited settings. Here, a closed‐tube, two‐stage, mixed‐dye‐based isothermal amplification method with ribonuclease‐cleavable enhanced probes (REP), termed REP‐TMAP, for dual‐visualization detection of SARS‐CoV‐2 variants including JN.1, BA.2, BA.4/5, and Delta is introduced. The first stage of REP‐TMAP is reverse transcription recombinase polymerase amplification and the second stage is dual‐visualization detection synergistically mediated by the REP and the mixed dyes of cresol red and hydroxy naphthol blue. In REP‐TMAP reaction, the color change under ambient light indicates SARS‐CoV‐2 infection, while the fluorescence change under blue light excitation specifies variant type. On detecting transcribed RNA of SARS‐CoV‐2 spike gene, this assay is rapid (within 40 min), highly sensitive (10–200 copies per reaction), and highly specific (identification of single‐base mutations). Furthermore, the assay has been clinically validated to accurately detect JN.1, BA.2, and BA.4/5 variants from 102 human oropharyngeal swabs. The proposed assay therefore holds great potentials to provide a rapid, dual‐visualization, sensitive, specific, point‐of‐care detection of SARS‐CoV‐2 variants and beyond.

## Graphical abstract

A closed‐tube, two‐stage, mixed‐dye‐based isothermal amplification method coupled with a ribonuclease‐cleavable enhanced probe (REP), termed REP‐TMAP, is developed for dual‐visualization detection of SARS‐CoV‐2 variants of interest. The color changes of REP‐TMAP reactions under ambient light indicate SARS‐CoV‐2‐positive infection, while the fluorescence changes under blue light excitation specify the variant types such as JN.1, BA.2, and BA.4/5.

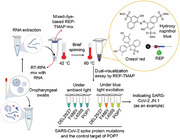

## 1 Introduction

The pandemic of coronavirus disease 2019 (COVID‐19) due to the spread of severe acute respiratory syndrome coronavirus 2 (SARS‐CoV‐2) has caused over 775 million infection‐confirmed cases and more than 7 million deaths worldwide as of 7 April 2024.^[^ ^1^ ^]^ Despite the World Health Organization (WHO) declaring an end to the global health emergency by COVID‐19, there remains a persistent threat of continuously emerging SARS‐CoV‐2 variants and subvariants with frequent mutations that can alter infectivity, transmissibility, and antigenicity.^[^ ^2^ ^]^ Therefore, timely assessment of variant transmission is still essential to combat the threat, particularly in resource‐limited settings, which necessitates a rapid, visual, sensitive, specific point‐of‐care (POC) technology capable of detecting SARS‐CoV‐2 variants.^[^ ^3^ ^]^

Currently, next generation sequencing (NGS) which can search whole viral genomes for unknown mutations is a dominant approach for accurate surveillance of SARS‐CoV‐2 variants or mutations.^[^ ^4^ ^]^ However, this method is rather time‐consuming (>3 d) and requires large‐scale, costly, and sophisticated instruments, as well as skilled professionals, limiting its POC applications.^[^ ^4^, ^5^ ^]^ In addition to NGS, reverse transcription polymerase chain reaction (RT‐PCR) followed by Sanger sequencing is another method frequently used for known‐mutations identification.^[^ ^6^ ^]^ Compared with NGS, the turnaround time of RT‐PCR plus Sanger sequencing can be narrowed down to one day, whereas its detection procedure is still time‐consuming (>4.5 h), including RT‐PCR, product purification, and Sanger sequencing.^[^ ^7^ ^]^ Resembling NGS, this routine method has to be finished using a sophisticated sequencer.^[^ ^8^ ^]^ Apart from Sanger sequencing, other strategies such as TaqMan probe,^[^ ^9^ ^]^ mass spectrometry,^[^ ^10^ ^]^ and melting curve analysis^[^ ^11^ ^]^ are also interfaced with RT‐PCR for SARS‐CoV‐2 variants detection. Unfortunately, the core RT‐PCR technology relies on benchtop thermal cycling equipment and requires approximately two‐hour assay time, unable to reach the goal of rapidly detecting SARS‐CoV‐2 variants detection.

As the alternative to RT‐PCR, isothermal nucleic acid amplification (INAA) without thermal cycling is promising to enable rapid, visual, sensitive, specific POC detection.^[^ ^12^ ^]^ Presently, two majors of INAA methods, recombinase polymerase amplification (RPA) and loop‐mediated isothermal amplification (LAMP), have been widely used.^[^ ^13^ ^]^ For years, our group has been pursuing the development of LAMP/RPA‐based POC technologies to improve their speed, sensitivity, and specificity.^[^ ^14^ ^]^ However, for direct mutation detection, RPA and LAMP are challenged by unrobust performance, multi‐step detection procedures, complicated primer design, and nonspecific signals.^[^ ^15^ ^]^ Thus, INAA‐based reliable mutation detection entails fluorescence resonance energy transfer (FRET)‐labeled oligonucleotide probes such as one‐step strand displacement probes,^[^ ^16^ ^]^ molecular beacons,^[^ ^17^ ^]^ cleavable beacon primer (CBP),^[^ ^18^ ^]^ and loop primer probe (LP).^[^ ^19^ ^]^ However, their actual performances vary from targets and probes, especially in visual mutation detection. Besides, the integration of INAA with CRISPR/Cas system (INAA‐CRISPR) is also documented for mutation detection. Whereas, robust INAA‐CRISPR calls for the optimization of guide RNA whose efficiency is tied to protospacer adjacent motif.^[^ ^20^ ^]^ Further, rapid visual INAA‐CRISPR assay entails much high concentrations of FRET‐labeled probes, causing unaffordable reagent cost.^[^ ^21^ ^]^ Therefore, an efficient, reliable, cost‐effective INAA method is demanded for SARS‐CoV‐2 variants visual detection.

In this study, we report a ribonuclease‐cleavable enhanced probe (REP)‐coupled, two‐stage, mixed‐dye‐based INAA assay, named REP‐TMAP, for rapid, closed‐tube, and dual‐visualization detection of SARS‐CoV‐2 variants. Differing from the ribonuclease‐cleavable probes reported previously,^[^ ^18^, ^19^ ^]^ this newly designed REP with an extended probe‐target hybridization region can enhance LAMP performance on discrimination of single‐base mutations. The dual‐visualization REP‐TMAP assay is achieved by combining the REP with the mixed dyes of cresol red (CR)^[^ ^22^ ^]^ and hydroxy naphthol blue (HNB).^[^ ^23^ ^]^ Under ambient light, the color change of REP‐TMAP reaction reflects SARS‐CoV‐2′s presence, whereas under blue light excitation, the fluorescence change identifies variant type. On detecting transcribed RNA of SARS‐CoV‐2 spike gene from JN.1, BA.2, BA.4/5, and Delta variants, our assay demonstrably possesses the advantages of rapidness (within 40 min), high sensitivity (10–200 copies per reaction), and high specificity (identifying single‐base mutation). Additionally, through testing 102 human oropharyngeal swabs, we clinically validate that the dual‐visualization REP‐TMAP assay can accurately detect JN.1, BA.2, and BA.4/5 variants and is comparable to the routine methods of RT‐PCR plus Sanger sequencing and RT‐qPCR. Thus, our proposed assay is efficient, reliable, and cost‐effective for POC visual detection of SARS‐CoV‐2 variants. Moreover, due to REP's programmability, we anticipate that the assay method is also applicable to detect other pathogens and variants, as well as genetically mutated biomarkers.

## 2 Results

### 2.1 The REP‐Coupled LAMP Enables Highly Specific Mutation Detection

**Figure 1:**
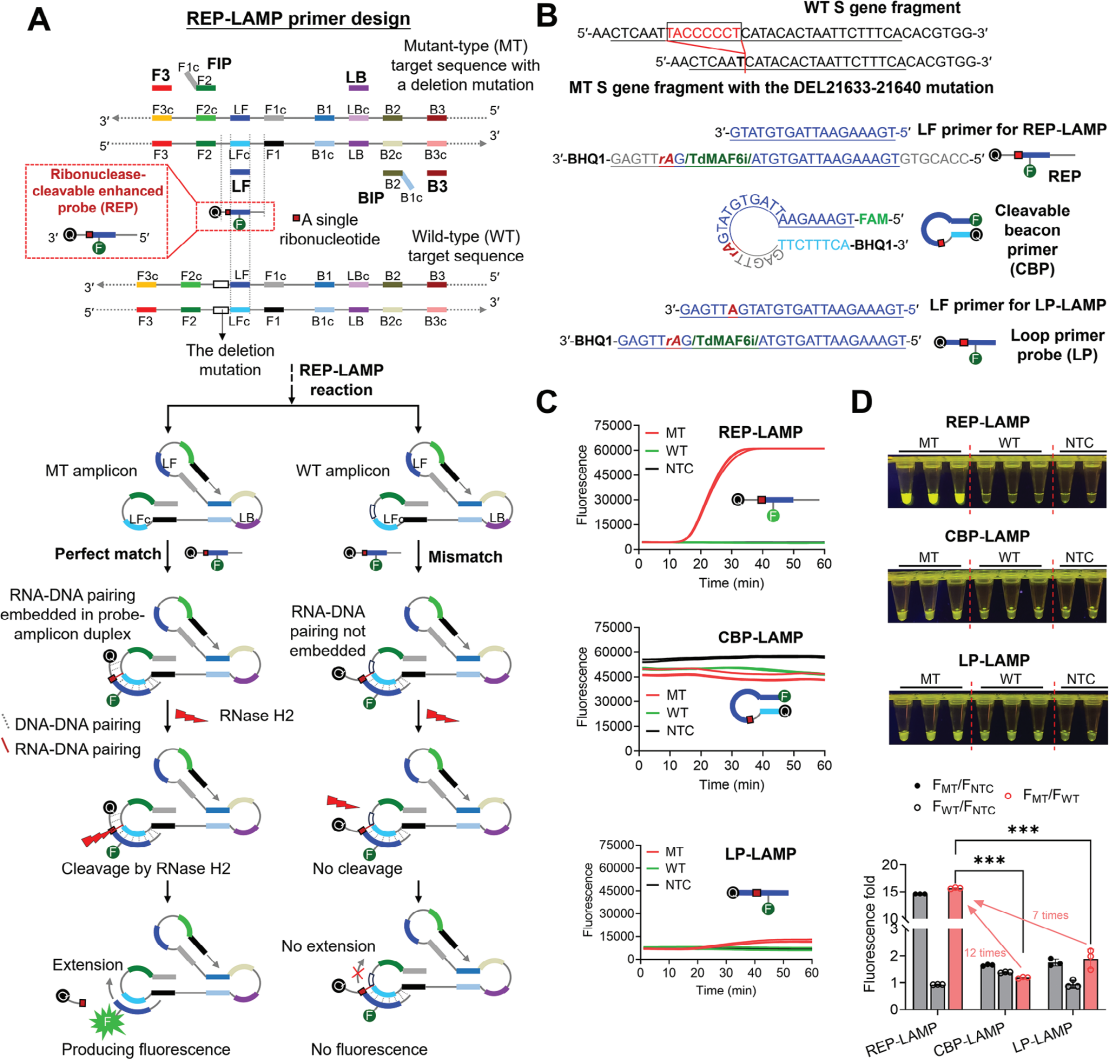
The principle of REP‐LAMP assay and its high specificity on mutation detection. A) Scheme of REP‐LAMP. The mutant‐type (MT) target sequence with a deletion mutation is only presented here. For the MT target sequence with a single base mutation, the scheme is shown in Figure S1 (Supporting Information). B) Sequences of WT target, MT target, the REP and its LF primer for REP‐LAMP, CBP, as well as the LP and its LF primer for LP‐LAMP. Although a short S gene fragment presents here, a pUC57 plasmid with or without the SARS‐CoV‐2 S gene mutation of DEL21633‐21640 (5′‐TACCCCCT‐3′) was used as the target. The bordered font is the mutation‐specific single ribonucleotide. C) Real‐time fluorescence detections of REP‐LAMP, CBP‐LAMP, and LP‐LAMP. Three replicates (n = 3) were run for each test. D) Endpoint visual detections and the comparison of fluorescence change fold. Each image of tube‐based visual detection is a representative of three independent experiments. Three replicates (n = 3) were run for each MT/ MT test, but two replicates (n = 2) for NTC tests. F/F_NTC_, the endpoint fluorescence intensity divided by that in NTC. MT, the positive reactions with 10^5^ copies of the pUC57 plasmids containing MT S gene fragment. WT, the positive reactions with 10^5^ copies of the pUC57 plasmids containing WT S gene fragment. NTC, no‐template control. Error bars represent the standard deviations of the three replicates. Unpaired two‐tailed *t*‐test was used to analyse significant difference between two groups. ***, *P* < 0.001.

To demonstrate the capability on mutation detection, we coupled the REP with LAMP to develop the REP‐LAMP assay. As shown by the red dashed frame in **Figure** **1**A, the REP is a linear oligonucleotide sequence that incorporates a single ribonucleotide. To constitute a FRET probe, the 3′ end deoxyribonucleotide and the nearest inner thymine (T) deoxyribonucleotide are labeled with quencher (Q) and fluorophore (F) groups, respectively, intentionally flanking the ribonucleotide. On sequence composition, differing from the reported ribonuclease‐cleavable probe for LAMP,^[^ ^18^, ^19^ ^]^ the REP typically comprises three parts: the entire LFc site (15–25 bp), the complete region (5–10 bp) between 3′ end of LFc and 5′ end of F1 sites, and the partial region (5–10 bp) between 3′ end of F2 and 5′ end of LFc sites. If REP recognizes the downstream target (i.e., the region between B1 and B2), the sequence composition is similar. Obviously, the REP possesses an enlarged hybridization region which probably promotes structural stability of the duplex formed by REP and its target (REP‐target duplex), particularly at LAMP's reaction temperatures (60–65 °C).

During REP‐LAMP reaction, the amplification products (amplicons) containing the sequences of mutant type (MT) or wild type (WT) in loops are accumulated exponentially (Figure 1A). After annealing, the REP perfectly matches the MT amplicon to form a ribonucleotide‐deoxyribonucleotide (RNA‐DNA) pair which is embedded in the probe‐amplicon duplex. However, the mismatching of REP and WT amplicon results in an open state at the end, forming a nonembedded RNA‐DNA pairing. When loading the RNase H2 which preferentially cleaves the ribonucleotide of RNA‐DNA pairing embedded in a DNA duplex, the perfectly matched REP is specifically cleaved by RNase H2, producing strong fluorescence. Meanwhile, a 3′ hydroxyl group in REP residues is produced to achieve primer‐like extension.^[^ ^24^ ^]^ However, for the WT amplicon, neither cleavage nor fluorescence happen, let alone primer‐like extension. Figure 1A only shows the scheme of REP‐LAMP to detect the MT target sequence with a deletion mutation. As to the MT target sequence with a single base mutation, the scheme of REP‐LAMP possesses some differences (Figure S1, Supporting Information). First, the mismatching of REP and WT amplicon can form the probe‐amplicon duplex, however, lacking the RNA‐DNA pairing due to single base mutation. Second, although REP is not cleaved by RNase H2 to produce fluorescence, the primer‐like extension may happen if the probe‐amplicon duplex with a DNA‐RNA unpairing is stable. Given this, we hypothesized that REP‐LAMP would be highly efficient for SARS‐CoV‐2 variants detection by targeting deletion and single‐base mutations.

To demonstrate the hypothesis, REP‐LAMP was applied to detect the S gene mutation of DEL21633‐21640 (5′‐TACCCCCT‐3′). Figure 1B shows the sequences of MT target, WT target, LF primer, REP, and the previously reported CBP and LP. The probes of REP, CBP and LP were all labeled using the FAM fluorophore and BHQ1 quencher. The optimized size of REP for the mutation detection was 31 bp, composed of 18 bp LF, the 7 bp region between 3′ end of LFc and 5′ end of F1, and the 6 bp region between 3′ end of F2 and 5′ end of LFc (Figure S2, Supporting Information). Furthermore, we found that a sensitive REP‐LAMP assay required the addition of LF primer (Figure S3A, Supporting Information). Then, we compared the optimized REP‐LAMP assay with previously reported CBP‐coupled LAMP (CBP‐LAMP) and LP‐coupled LAMP (LP‐LAMP) assays.^[^ ^18^, ^19^ ^]^ As shown in Figure 1C, in real‐time fluorescence detection at 60 °C, exponential fluorescence curves present in the REP‐LAMP and LP‐LAMP assays with MT targets. However, due to structural instability of hairpin at 60 °C (Figure S4, Supporting Information), the linearized CBP widens the distance between FAM and BHQ1, causing high background fluorescence. Accordingly, as depicted in Figure 1C, CBP‐LAMP is not suitable for real‐time fluorescence mutation detection.^[^ ^18b^ ^]^ In contrast, LP‐LAMP is feasible to identify MT targets.^[^ ^19^ ^]^ Unfortunately, the fluoresce changes in LP‐LAMP are much lower than those in REP‐LAMP assays (Figure 1C), likely due to insufficient probe‐target hybridization. Previously, CBP‐LAMP and LP‐LAMP were also proved to perform endpoint fluorescence detection. Thus, we further compared their endpoint detection performances. As depicted in Figure 1D, the REP‐LAMP with MT target achieves the best visual detection with the highest fluorescence fold (F_MT_/F_WT_), ≈12‐time (7‐time) higher than the CBP‐LAMP (LP‐LAMP) with MT targets. Furthermore, the products of REP‐LAMP assays with and without the LF primer were analyzed by using denaturing polyacrylamide gel electrophoresis. As shown in Figure S3B (Supporting Information), REP is only cleaved in the RNase H2‐loaded LAMP assay with MT targets and subsequently it turns into various sizes of extended residues due to primer extension, indirectly proving the principle of REP‐LAMP reaction. Therefore, our REP‐LAMP assays are evidently superior to currently reported methods (i.e., CBP‐LAMP and LP‐LAMP assays) in both real‐time and endpoint visual mutation detections.

### 2.2 Mixed‐Dye‐Based REP‐LAMP for Target DNA and Its Mutation Detection

Endpoint visual REP‐LAMP assays are typically carried out according to the intensity changes of single‐color fluorescence (Figure 1D). However, such changes involve the preset of cutoff values and are invisible under ambient light. A dual‐visualization assay with color changes under both ambient light and light excitation are therefore anticipated to facilitate POC detection. To this end, we added the mixed dyes of CR and HNB into REP‐LAMP reaction to develop a mixed‐dye‐based REP‐LAMP method for dual‐visualization detection of target DNA and its mutation.

**Figure 2:**
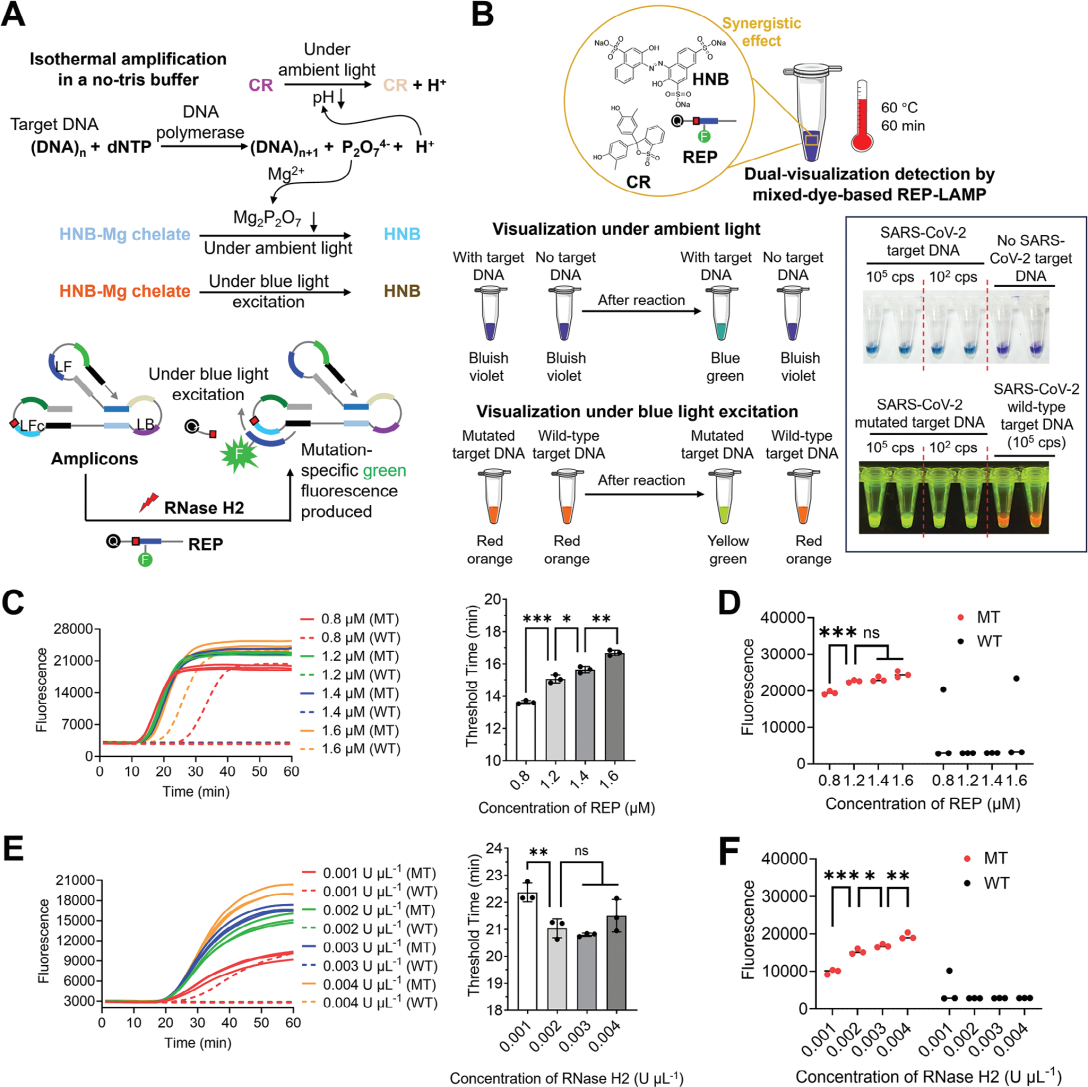
Dual‐visualization assay by mixed‐dye‐based REP‐LAMP for target DNA and its mutation. A) Biochemical reactions in the assay. B) Scheme of dual‐visualization detection of target DNA and its mutation using the synergistic effect of HNB, CR, and REP. Target DNA and its mutation were the 10^5^ or 10^2^ copies (cps) of the pUC57 plasmids containing the SARS‐CoV‐2 S gene fragment with the mutation of DEL21633‐21640 (5′‐TACCCCCT‐3′). Two replicates (n = 2) were run for each test. Each image of tube‐based visual detection is a representative of three independent experiments. C) Effect of REP on real‐time fluorescence detection and threshold time comparison among the reactions with mutated target (MT) DNA. D) Effect of REP on endpoint fluorescence detection. E) Effect of RNase H2 on real‐time fluorescence detection and time comparison among the reactions with mutated target (MT) DNA. F) Effect of RNase H2 on endpoint fluorescence detection. Mutated target (MT) DNA, 10^5^ copies of the pUC57 plasmids with the S gene mutation. Wild‐type target (WT) DNA, 10^5^ copies of the pUC57 plasmids with the wild‐type S gene fragment. Three replicates (n = 3) were run for each test. Error bars represent the standard deviations of the three replicates. Unpaired two‐tailed *t*‐test was used to analyse significant difference between two groups. ***, *P* < 0.001; **, *P* < 0.01; *, *P* < 0.05; *ns*, not significant.

As shown in **Figure** **2**A, in a no‐tris buffered isothermal amplification reaction, DNA polymerase‐mediated primer extension (i.e., DNA replication) exponentially consumes dNTPs to produce hydrogen ions (H^+^) and pyrophosphate ions (P_2_O_7_^4−^). Accumulated H^+^ decreases the reaction solution's pH while the P_2_O_7_^4−^ can precipitate free Mg^2+^ to form Mg_2_P_2_O_7_.^[^ ^25^ ^]^ Consequently, under ambient light, CR senses the pH decreasing with a color change from fuchsia to yellow (Figure S5A, Supporting Information), while HNB enables a color change from light blue to sky blue as HNB‐Mg chelates continue to decline (Figure S5B, Supporting Information). Jointly, the mixed dyes of CR and HNB make the REP‐LAMP reaction to perform a color change from bluish violet to blue green under ambient light (Figure S5C, Supporting Information). On the other hand, under the blue light excitation, only the HNB‐Mg chelates emit a single red fluorescence with decreased intensity after amplification (Figure S5B and C, Supporting Information). Interestingly, when using a FAM‐labeled REP, the cleavage of mutation‐specific REP by RNase H2 generates strong green fluorescence under blue light excitation (Figure 2A). Accordingly, the synergistic effect of HNB, CR, and REP enables dual‐visualization detection of target DNA and its mutation, based on color changes under ambient light and blue light excitation (Figure 2B).

To verify this capability, the mixed‐dye‐based REP‐LAMP was applied to the dual‐visualization detection of SARS‐CoV‐2 S gene DNA and its mutation of DEL21633‐21640 (5′‐TACCCCCT‐3′). As shown in Figure 2B, the color change from bluish violet to blue‐green (under ambient light) reveals whether there is a SARS‐CoV‐2 target DNA, while the fluorescence change from red orange to yellow green (blue light excitation) indicates the type of mutation. Note that the selectivity of light excitation types greatly depends on two factors: the properties of fluorophore in the REP and the fluorescence color in negative reactions. In this study, the fluorescence of negatives is red, and the FAM is used in REP; therefore, the blue light excitation is chosen to achieve dual‐visualization detection.

Next, the concentrations of REP and RNase H2 were optimized to achieve a robust dual‐visualization detection. In real‐time fluorescence detections, raising REP's concentration (from 0.8 to 1.6 µM) substantially retards the reactions with MT targets due to increasing threshold time (Figure 2C). Moreover, when applying 0.8 or 1.6 µM REP, some false positives are occasionally induced in reactions with WT targets (Figure 2C; Figure S6A, Supporting Information). For endpoint fluorescence, adding more than 1.2 µM REP did not significantly increase the fluorescence and only 1.2 or 1.4 µM REP enabled robust reactions with WT targets (Figure 2D). Thus, considering visual detection robustness and the cost, we chose 1.2 µM REP as the optimal one. As the core enzyme to cleave REP, RNase H2 with 0.002 U µL^−1^ or more is not beneficial to boost the amplification, but remarkably strengthens the fluorescence (Figure 2E and F). From the cost analysis, however, 0.002 U µL^−1^ RNase H2 is the best to realize reliable and cost‐effective dual‐visualization detection (Figure 2F; Figure S6B, Supporting Information). Thus, a robust dual‐visualization DNA detection can be implemented using 1.2 µM REP and 0.002 U µL^−1^ RNase H2.

### 2.3 Dual‐Visualization Detection of Target RNA and Its Mutation by REP‐TMAP

Based on mixed‐dye‐based REP‐LAMP, we successfully developed dual‐visualization detection of target DNA and its mutation. However, SARS‐CoV‐2 is an RNA virus^[^ ^26^ ^]^ and thus, the establishment of RNA detection is indispensable. To develop such method, in vitro transcribed RNA sequences corresponding to the S gene of SARS‐CoV‐2 BA.4/5, BA.2, and Delta were prepared as templates.

First, by directly adding reverse transcriptase and RNA targets (Figure S7A, Supporting Information), we assessed the performance of one‐pot, single‐stage mixed‐dye‐based REP‐LAMP. As shown in Supporting Information section (Figure S7B–D, Supporting Information), except for BA.4/5, the dual‐visualization assays only detect down to 10^4^ copies of S gene RNA targets per reaction for both Delta and BA.2 within 60 min, the same as those of real‐time fluorescence assays (Figure S8, Supporting Information). Obviously, single‐stage mixed‐dye‐based REP‐LAMP possesses insufficient sensitivity, likely resulting from the inhibited reverse transcription (RT) processes by RNase H2 which literately influences DNA‐RNA pairing.^[^ ^24b^ ^]^

**Figure 3:**
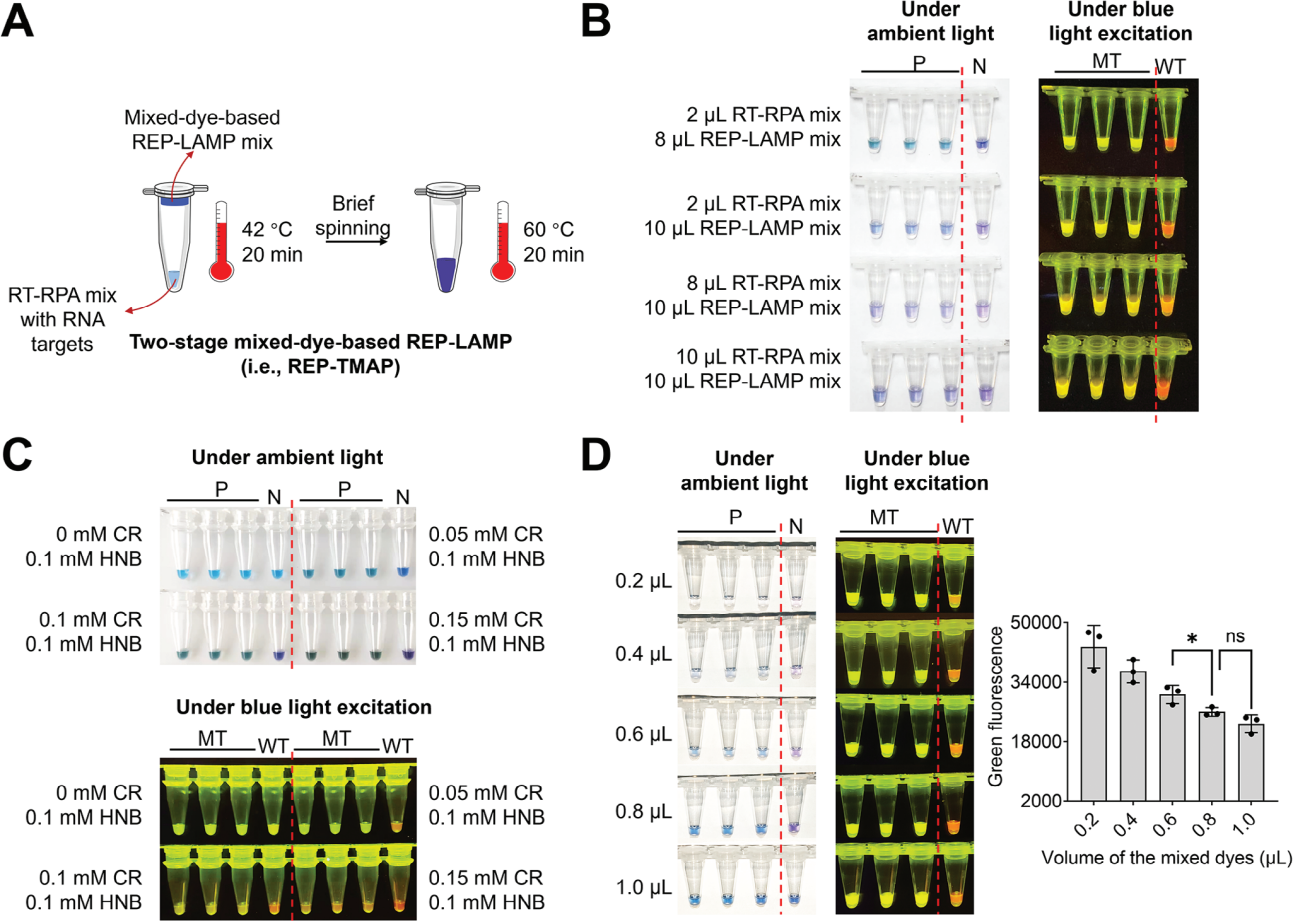
Dual‐visualization REP‐TMAP assays for detecting transcribed SARS‐CoV‐2 S gene RNA and its mutation. A) Workflow of the dual‐visualization detection by REP‐TMAP. B) Effect of the volume ratios of the RT‐RPA mix to the mixed‐dye‐based REP‐LAMP mix on the dual‐visualization detection. C) Effect of the ratios of CR to HNB dyes in the mixed dyes. D) Effect of the volumes of the mixed dyes at an optimal ratio. P and MT, the positive (P) reactions with 10^5^ copies of transcribed mutation‐type (MT) S gene RNA. The mutation is the DEL21633‐21640 (5′‐TACCCCCT‐3′). N, the negative reactions without SARS‐CoV‐2 S gene RNA. WT, the reactions with 10^5^ copies of transcribed wild‐type (WT) S gene RNA. Each image of tube‐based visual detection is a representative of three independent experiments. Three replicates (n = 3) were run for each positive test. Error bars represent the standard deviations of the three replicates. Unpaired two‐tailed *t*‐test was used to analyse significant difference between two groups. *, *P* < 0.05; *ns*, not significant.

To circumvent the RT inhibition by RNase H2, we came up with a two‐stage mixed‐dye‐based REP‐LAMP assay, namely REP‐TMAP which combined RT‐RPA reaction and mixed‐dye‐based REP‐LAMP reaction in a closed tube. As shown in **Figure** **3**A, the RNA target‐containing RT‐RPA mix on the tube's bottom initiates the first‐stage reaction (42 °C for 20 min) and the mixed‐dye‐based REP‐LAMP mix on the tube's lid executes the second‐stage reaction (60 °C for 20 min) after brief spinning. To develop a robust dual‐visualization detection, we first optimized the volume ratio of RT‐RPA mix to REP‐LAMP mix. As shown in Figure 3B, supporting more RT‐RPA mix apparently weakened the dual‐visualization performance, especially the color change under ambient light. Since RT‐RPA reaction comprises tris buffer and MgOAc,^[^ ^27^ ^]^ the high amount of the mix influences the color change of both CR and HNB under ambient light, thereby decreasing dual‐visualization performance. Thus, the best combination for dual‐visualization detection is 2 µL RT‐RPA mix and 8 µL of the mixed‐dye‐based REP‐LAMP mix.

Then, the ratio of CR and HNB in the mixed dyes were optimized. Figure 3C shows that 0.05 mM CR and 0.1 mM HNB could mediate the best dual‐visualization performance. Furthermore, the mixed‐dye amount at the optimal ratio was investigated by tuning the volume. As revealed in Figure 3D and Figure S9 (Supporting Information), increasing the volume from 0.2 to 0.8 µL remarkably reduces the green fluorescence from FAM fluorophore, while having minimal impact on dual‐color fluorescence change under blue light excitation. However, for color changes under ambient light, the volume of 0.8 µL brings about the best distinguishable visualization. Contrarily, adding 1.0 µL weakens the distinguishability of color change, likely because that the excess of HNB can disable visualization effect when observed by naked eyes.^[^ ^23^ ^]^ Together, 0.8 µL of the mixed dyes (i.e., 0.04 mM CR and 0.08 mM HNB in reaction) is optimal to develop the robust dual‐visualization REP‐TMAP assay in which the positive reactions present blue‐green color under ambient light, while the positive reactions with MT targets emit yellow‐green fluorescence under blue light excitation.

### 2.4 Performance of Dual‐Visualization REP‐TMAP Assay for SARS‐CoV‐2 Variants of Interest

**Figure 4:**
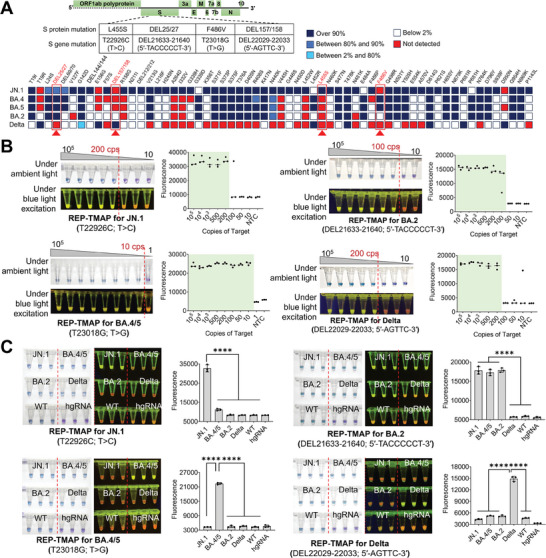
Sensitivity and specificity of dual‐visualization REP‐TMAP assays for the detection of four SARS‐CoV‐2 variant of interest (JN.1, BA.2, BA.4/5, and Delta). A) SARS‐CoV‐2 genome map indicating the S protein/gene mutations specific to SARS‐CoV‐2 variants JN.1, BA.2, BA.4/5, and Delta. The lineage comparison inset was captured from GISAID. B) Sensitivities and the endpoint fluorescence comparison of the assays for various copies (cps) of JN.1, BA.2, BA.4/5, and Delta S gene RNA. C) Specificities and the endpoint fluorescence comparison of the assays. hgRNA, human genomic RNA. All the targets with 10^4^ copies (cps) were loaded into each reaction. Each image of tube‐based visual detection is a representative of three independent experiments. Three replicates (n = 3) were run for each test. Error bars represent the standard deviations of the three replicates. Unpaired two‐tailed *t*‐test was used to analyse significant difference between two groups. ****, *P* < 0.0001.

Given the superiority of mutation detection, the dual‐visualization REP‐TMAP assay was leveraged to detect SARS‐CoV‐2 variants JN.1, BA.2, BA.4/5, and Delta. As shown in **Figure** **4**A, the S gene mutations of T22926C (T > C; single‐base mutation), DEL21633‐21640 (5′‐TACCCCCT‐3′; deletion mutation), T23018G (T > G; single‐base mutation), and DEL22029‐22033 (5′‐AGTTC‐3′; deletion mutation) were selected as the targets. According to the lineage comparison provided by GISAID,^[^ ^28^ ^]^ these four S gene mutations code the S protein mutations of L455S, DEL25/27, F486V, and DEL157/158 with more than 80% prevalence in SARS‐CoV‐2 variants JN.1, BA.2, BA.4/5, and Delta, respectively. Note that the DEL25/27 mutation is also shared by JN.1 and BA.4/5. Using the optimized reaction above and the templates of transcribed S gene RNA, we evaluated the dual‐visualization assay sensitivity and specificity.

As shown in Figure 4B, attributed to RT‐RPA‐based preamplification, the sensitivity of dual‐visualization REP‐TMAP assays within 40 min is significantly improved by two orders of magnitude, consistently detecting down to 200, 100, 10, and 200 copies of JN.1, BA.2, BA.4/5, and Delta S gene RNA, respectively. Furthermore, real‐time fluorescence REP‐TMAP assays achieve the same sensitivities (Figure S10, Supporting Information). To evaluate the specificity, the human genomic RNA and the S gene RNAs of SARS‐CoV‐2 JN.1, BA.2, BA.4/5, Delta, and WT were tested in each mutation‐specific dual‐visualization assay simultaneously. Obviously, under ambient light, the color change of REP‐TMAP reaction indicates the presence of SARS‐CoV‐2 RNA, whereas the visual detection under blue excitation identifies the variant type (Figure 4C). Also, real‐time fluorescence assays possess the same specificities without any cross‐reactivity (Figure S11, Supporting Information). As a result, our REP‐TMAP assay is highly sensitive and specific for rapid and dual‐visualization detection of S gene RNA targets from SARS‐CoV‐2 variants JN.1, BA.2, BA.4/5, and Delta.

### 2.5 Clinical Validation of the Dual‐Visualization REP‐TMAP Assay

**Figure 5:**
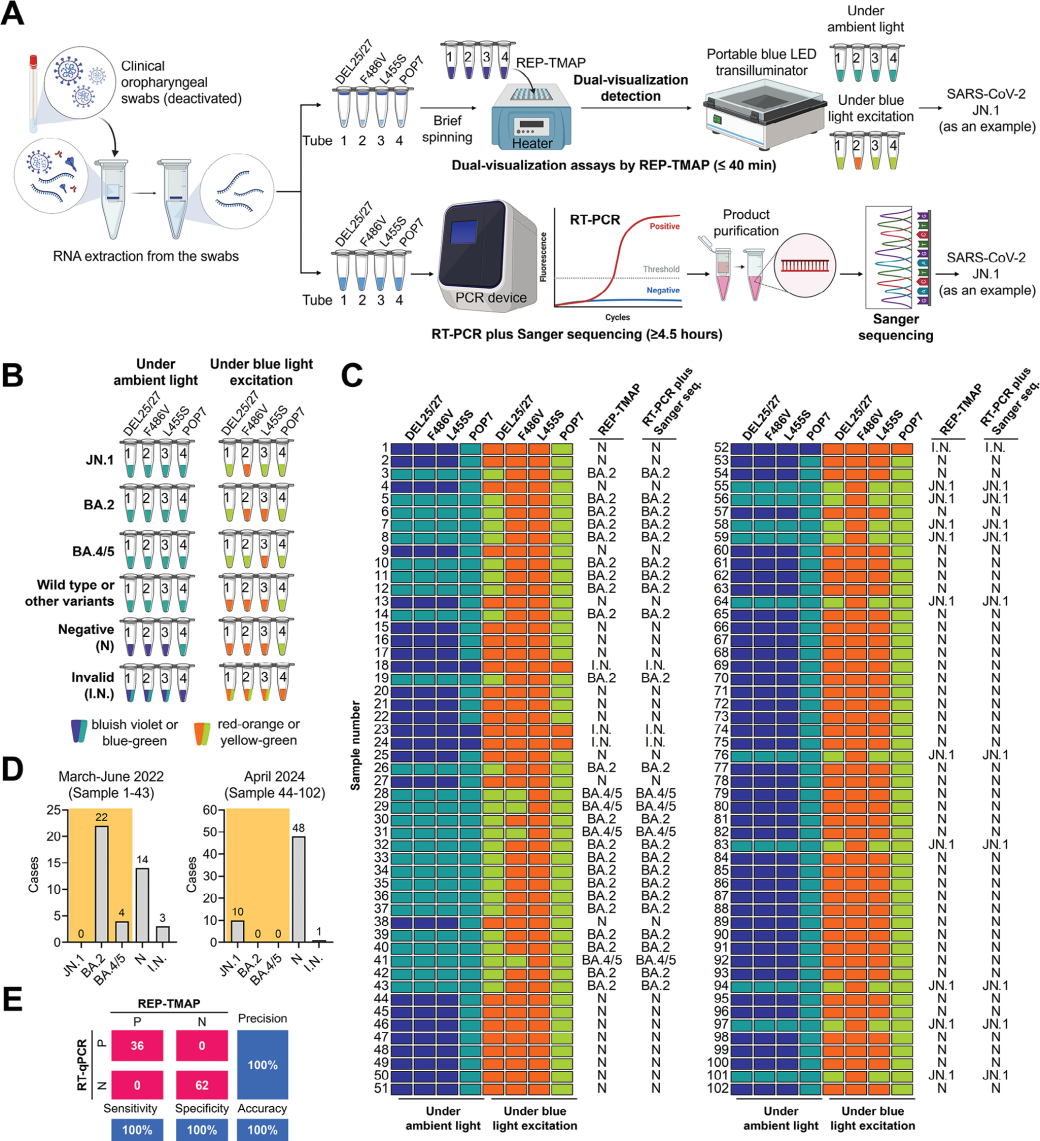
Clinical sample testing of dual‐visualization REP‐TMAP assay on detecting three SARS‐CoV‐2 variants of interest (JN.1, BA.2, and BA.4/5). A) Workflow of SARS‐CoV‐2 variant detection in clinical oropharyngeal swabs when using the dual‐visualization assay and the routine RT‐PCR plus Sanger sequencing. Tube 1–4, the tests to identify the SARS‐CoV‐2 S gene mutations associated with S protein mutations of DEL25/27, F486V, and L455S, as well as the POP7 control gene. The JN.1 detection was used as an example. hgRNA, human genomic RNA. Created with BioRender.com. B) Criterion to judging JN.1, BA.2, BA.4/5, wild type/other variant types, negative, and invalid, based on the dual‐visualization assay results. C) Dual‐visualization assay results of 102 samples (Sample 1–102) for each mutation and the variant identification results by RT‐PCR plus Sanger sequencing. D) Distribution of SARS‐CoV‐2 variants (shaded in color) at two different time periods (Sample 1–43 in March‐June 2022 and Sample 44–102 in April 2024). E) Confusion matrix describing the overall performances of the REP‐TMAP and the commercial RT‐qPCR assays between positive and negative samples. N, negative; I.N., invalid.

As of now, SARS‐CoV‐2 Omicron has become the main variant in China.^[^ ^29^ ^]^ Thus, we dismissed the screening of Delta by focusing on the Omicron subtype JN.1, BA.2, and BA.4/5 variants detection. Using a commercial RNA extraction kit, we first extracted RNA from clinical oropharyngeal swabs (deactivated) which were collected from 102 volunteers (**Figure** **5**A). Subsequently, we quartered each sample's RNA extract and loaded it into four tubes (Tube 1–4) for closed‐tube, two‐stage dual‐visualization REP‐TMAP assays within 40 min. The RNA extract in each tube was mixed with a low‐volume RT‐RPA mixture at the bottom to initiate the first‐stage reaction, and then, after brief spinning, the centrifuged mixed‐dye‐based REP‐LAMP mixture on the lid triggered the second‐stage reaction. As shown in Figure 5A, Tube 1–3 contained the REP‐TMAP reagents specific to the S gene mutations of DEL21633‐21640 (5′‐TACCCCCT‐3′), T23018G (T > G), and T22926C (T > C), which referred to the S protein mutations of DEL25/27, F486V, and L455S, respectively. The REP‐TMAP reagent targeting human POP7 gene in Tube 4 was the quality control of RNA extraction. To meet POC application potential, all the reactions were incubated in a mini metal incubator and the detection was performed using a portable blue LED transilluminator.

To better understand the test, one sample infected by SARS‐CoV‐2 JN.1 is taken as an example. Thus, a valid RNA extract should contain both JN.1 RNA and human genomic RNA. Since the color change under ambient light can be mediated by any target‐triggered LAMP reactions, this visualization assay is only applicable for detecting targets, not for differentiating target types. Thus, Tube 1–4 all present blue‐green under ambient light, hinting SARS‐CoV‐2 positive. Meanwhile, because that JN.1 RNA has the mutations of DEL25/27 and L455S (Figure 4A), Tube 1 and 3 emit yellow‐green fluorescence under blue light excitation due to the cleavage of REP. Besides, Tube 4 also generates yellow‐green fluorescence since human genomic RNA carries the POP7 gene to trigger REP‐TMAP reaction. Together, yellow‐green fluorescence in Tube 1, 3, and 4 under blue light excitation indicates the variant type of JN.1. Figure 5B shows a criterion to the result judgement of dual‐visualization REP‐TMAP assays. If some results cannot be judged with the criterion, they likely belong to other variants, which need further investigation. As a comparative analysis, SARS‐CoV‐2 variants detection was also conducted using the routine RT‐PCR plus Sanger sequencing method which usually takes over 4.5 h.

As displayed in Figure 5C and Figure S12 (Supporting Information), the visualization assay result under ambient light revealed a SARS‐CoV‐2‐positive rate of 35.3% (36/102) and a SARS‐CoV‐2‐negative rate of 59.8% (61/102). Also, 3.9% (4/102) were invalid due to POP7‐negative results, likely associated with RNA extraction failure. According to the visualization result under blue light excitation (Figure 5C), 27.8 (10/36), 61.1% (22/36), and 11.1% (4/36) of the SARS‐CoV‐2‐positive samples were identified to be variants JN.1, BA.2, and BA.4/5, respectively. Since the swabs were sampled at two different time periods (No. 1–43 in March‐June 2022 and No. 44–102 in April 2024), it's necessary to compare the changes of variant types as time. As shown in Figure 5D, in March‐June 2022, BA.2 was the dominant variant (84.6%, 22/26), BA.4/5 only accounted for 15.4% (4/26), while JN.1 was yet to be evolved. This prevalence trend was similar as what reported by the Chinese CDC at the time of January‐June 2022.^[^ ^29^ ^]^ In April 2024, although a SARS‐CoV‐2‐positive rate of 16.9% (10/59) was indicated (Figure 5D), all the positive samples were infected by JN.1, the dominant strain currently circulating in China.^[^ ^30^ ^]^ Thus, our dual‐visualization assay can be used for SARS‐CoV‐2′s continuing evolution assessment.

Upon variant identification, the dual‐visualization REP‐TMAP assay reached a 100% agreement with the RT‐PCR plus Sanger sequencing (Figure 5C and Table S1, Supporting Information). As for SARS‐CoV‐2 detection, our assays achieved 100% (36/36) sensitivity and 100% (62/62) specificity if focusing on all the valid sample tests, compared with a commercial RT‐qPCR kit approved by National Medical Products Administration (NMPA) of China (Figure 5E; Figure S13 and Table S2, Supporting Information). Thus, without large‐scale instruments, our method offers a rapid, simple, cost‐effective, and dual‐visualization SARS‐CoV‐2 variants detection.

## 3 Discussion

In summary, the present study reveals that the newly designed REP with an extended hybridization region can significantly improve the performance of LAMP‐based real‐time fluorescence and endpoint mutation detection (Figure 1C). Moreover, the REP with the mixed dyes of CR and HNB can mediate the color changes of reactions under both ambient light and blue light excitation (Figure 2). Based on such findings, we successfully develop a rapid, efficient, reliable, cost‐effective, dual‐visualization REP‐TMAP assays (Figure 3). The assays are also validated to identify three SARS‐CoV‐2 variants of interest, JN.1, BA.2, and BA.4/5 from 102 clinical oropharyngeal swabs (Figure 5).

Currently, although ribonuclease‐cleavable probes‐coupled LAMP assays (e.g., CBP‐LAMP and LP‐LAMP) can realize mutation detection,^[^ ^18^, ^19^ ^]^ their performances vary significantly, particularly in endpoint visual detection of RNA mutations. In this study, we propose a novel REP design featuring an expanded hybridization region (from 25–45 bp) to enhance the stability of probe‐target annealing, thereby remarkably improving the LAMP performance in endpoint visual mutation detection. Based on REP‐LAMP, we develop the dual‐visualization REP‐TMAP method for SARS‐CoV‐2 RNA detection and its mutation identification. The color change under ambient light readily indicates the presence of SARS‐CoV‐2 infection and meanwhile, the dual‐color fluorescence change visually identifies the type of mutation/variant (Figure 3 and Figure 4). To our knowledge, it is the first report of dual‐visualization‐based SARS‐CoV‐2 mutation/variant detection using closed‐tube isothermal amplification.

To develop a robust dual‐visualization assay, it is crucial to optimize the volume ratio of RT‐RPA mix to mixed‐dye‐based REP‐LAMP mix. Besides, the overall dual‐visualization performance is also influenced by the amount and ratio of CR to HNB in the mixed dyes, as well as the concentrations of REP and RNase H2. In this study, a robust dual‐visualization REP‐TMAP assay requires 1.2 µM REP, 0.002 U µL^−1^ RNase H2, 2 µL RT‐RPA mix, 8 µL of the mixed‐dye‐based REP‐LAMP mix, 0.04 mM CR, and 0.08 mM HNB. On detecting artificial RNA target of SARS‐CoV‐2 S gene, the assay exhibits high sensitivity (10–200 copies per reaction) and high specificity (identifying single‐base mutation) in less than 40 min. On clinical sample testing, our dual‐visualization assay results are consistent with those from the RT‐PCR plus Sanger sequencing and the commercial RT‐qPCR assay. Compared with reported isothermal amplification strategies for SARS‐CoV‐2 nucleic acid detection (Table S3, Supporting Information), REP‐TMAP is a rapid, simple, cost‐efficient (Table S4, Supporting Information), sensitive, and specific method to realize dual‐visualization‐based SARS‐CoV‐2 detection and its variant identification.

Certainly, the REP‐TMAP assay holds great potentials for further expansion and improvement in future studies. First, the detection throughput will be enhanced if replacing the first‐stage RT‐RPA with a multiplexed RT‐RPA.^[^ ^31^ ^]^ Second, the assay can be interfaced with microfluidic platforms (e.g., 3D‐printed lab‐on‐disc chips and pH‐paper‐based extraction system)^[^ ^32^ ^]^ and 2D nanocrystals (e.g., graphene and MoS_2_),^[^ ^33^ ^]^ thus streamlining manual operations and enabling POC optical/electrical biosensors, respectively. Third, to provide a mini but smart POC testing platform, portable chip devices can be integrated with rapid sample preparation, REP‐TMAP reaction, real‐time fluorescence detection, and smartphone‐based result analysis.^[^ ^34^ ^]^ Last, due to the REP's programmability, our dual‐visualization REP‐TMAP assay is readily adapted for emerging SARS‐CoV‐2 variants detection (e.g., SARS‐CoV‐2 JN.1.13 and JN.1.18),^[^ ^35^ ^]^ other infectious pathogens and their variants detection (e.g., Monkeypox and multidrug‐resistant Mycobacterium tuberculosis),^[^ ^36^ ^]^ the early cancer diagnostics associated with mutated nucleic acid biomarkers (e.g., the mutated *KRAS* gene),^[^ ^37^ ^]^ and more.

## 4 Experimental Section

### Materials

All the used plasmids, primers, and probes were purchased from Sangon Biotech (China). The sequence information was listed in Tables S5 and S6 (Supporting Information). MgSO_4_ (Cat# B1003S), dNTPs (Cat# N0447L), *Bst* 2.0 WarmStar DNA polymerase (Cat# M0538L), WarmStart RTx (Cat# M0380S), ET SSB (Cat# M2401S), and nuclease‐free water (Cat# B1500S) were purchased from New England Biolabs (NEB, USA). RNase H (Cat# D7089), 30% Acr‐Bis (29:1) (Cat# ST003), TEMED Substitute (Cat# ST728), ammonium persulfate substitute (Cat# ST005), urea (Cat# ST1731), and 6× DNA loading buffer (Cat# D0071) were purchased from Beyotime Biotech (China). SuperScript IV RT (Cat# 18 090 050) was purchased from Thermo Fisher Scientific (USA). RNase H2 was purchased from Integrated DNA Technologies (IDT, USA). The 10× Low buffer (100 mM (NH_4_)_2_SO_4_, 500 mM KCl, 20 mM MgSO_4_, 1% Tween 20, 1.0 M KOH, and pH 8.5 at 25 °C) was prepared in the laboratory. T7 High Yield RNA Transcription kit (Cat# TR101‐01), HiScript II One Step RT‐PCR Kit (Cat# P611‐01), and AceQ Universal SYBR qPCR Master Mix (Cat# Q511‐02) were purchased from Vazyme Biotech (China). Universal DNA purification kit (Cat# DP214‐02), RNA clean kit (Cat# DP412) and 10 000 × GeneRed Nucleic acid dye (Cat# RT211) were purchased from Tiangen Biotech (TIANGEN, China). The TwistAmp Basic kit (Cat# TABAS03KIT) for RPA was purchased from TwistDX (TwistDX, UK). QIAamp Viral RNA mini kit (Cat# 52 904) was purchased from QIAGEN Biotech (QIAGEN, USA). Commercial COVID‐19 Coronavirus Real Time PCR Kit (Cat# JC10223‐1N) was purchased from BioPerfectus Biotech (China). The 5× TBE buffer was purchased from Sangon Biotech. The 20× EvaGreen (Cat# 31 000) was purchased from Biotium Biotech (Biotium, USA). Cresol red (Cat# C6560) was purchased from Solarbio Life Sciences (China). Hydroxy naphthol blue (Cat# H810857) was purchased from Macklin Biotech (China). Disposable virus sampling tubes (Cat# ZY‐BDG‐3 mL) were purchased from Jiangsu Zhiyu Medical Equipment Co., Ltd (ZYMM, China).

### Ethics Statement

All clinical oropharyngeal swabs from volunteers were collected with a protocol approved by the ethics committee at the Zhongda Hospital of Southeast University (Protocol # 2023ZDSYLL325‐P01). The sampling process was carried out in strict accordance with the prescribed standards and all volunteers’ informed consents were obtained.

### Synthetic Nuclease Acid Targets

The S gene sequences of SARS‐CoV‐2 variants Delta, JN.1, BA.2, and BA.4/5 were downloaded from the National Center Biotechnology Information (NCBI) (https://www.ncbi.nlm.nih.gov/). The pUC57 plasmids inserted with the specific S gene sequences were all synthesized by Sangon Biotech. The S gene RNA targets were prepared in the laboratory using the T7 High Yield RNA Transcription kit. Briefly, the 40 µL reaction mixture was incubated at 37 °C for 16 h using TGreat Gradient Thermal Cycler (TIANGEN), then 2 µL DNaseI was added for 2 h incubation at 37 °C to eliminate residual DNA target. The RNA purification step was conducted using the RNA clean kit. The purified RNA template using Nano Drop (Thermo Fisher Scientific) to determine nucleic acid concentration and stored at −80 °C.

### Design of Primers and Probes

All the used LAMP primers (F3, B3, FIP, BIP, LF, and LB) in the study were designed using the online PrimerExplorer tool (https://primerexplorer.jp/e/). The *in‐silico* screening of primers and probes were run by using the online tools of Nucleic Acid Package (NUPACK, https://old.nupack.org/) and Oligonucleotide Properties Calculator (Oligo Calc, http://biotools.nubic.northwestern.edu/OligoCalc.html). The used probes of CBP, LP and REP were all designed to target the forward loop region, and each was inserted with a mutation‐specific ribonucleotide. The optimal forms of CBP and LP were designed according to what reported previously.^[^ ^18^, ^19^ ^]^ By targeting the LFc site, the sequence of REP should contain the entire LFc site (15–25 bp), the complete region (5–10 bp) between 3′ end of LFc and 5′ end of F1 sites, as well as a partial region (5–10 bp) between 3′ end of F2 and 5′ end of LFc sites. To construct a FRET probe, the 3′ end deoxyribonucleotide and the adjacent inner thymine deoxyribonucleotide of REP were intentionally labeled with BHQ1 quencher and FAM fluorophore groups, respectively, strategically flanking the mutation‐specific ribonucleotide.

### REP‐LAMP Assays

In the assays, the pUC57 plasmid containing the S gene mutation of DEL21633‐21640 (5′‐TACCCCCT‐3′) was used as the template, which was associated with the S protein mutation (DEL25/27) of SARS‐CoV‐2 variant BA.2. The optimized REP‐LAMP reaction mixture (10 µL) contained 1× Low Buffer, 4 mM MgSO_4_, 1.6 mM each dNTPs, 0.2 µM F3, 0.2 µM B3, 1.6 µM FIP, 1.6 µM BIP, 0.8 µM LF, 0.8 µM LB, 1.2 µM REP, 0.32 U µL^−1^ *Bst* 2.0 WarmStart DNA polymerase, 0.002 U µL^−1^ RNase H2, 1 µL of target DNA (10^5^ copies µL^−1^), and nuclease‐free water. The reactions were incubated at 60 °C for 60 min and the fluorescence signal was measured at 60 s intervals in a real‐time fluorescent quantitative PCR instrument (Archimed X4, ROCGENE, China). After amplification, the endpoint fluorescence imaging was conducted using a portable blue LED transilluminator (TIANGEN, China) and the images were captured with a smartphone.

### Dual‐visualization Assays of Mixed‐dye‐based REP‐LAMP

The mixed dyes of HNB and CR were added into the optimized REP‐LAMP reaction to achieve the dual‐visualization assay. The reaction system (10 µL) contained 1× Low Buffer, 4 mM MgSO_4_, 1.6 mM each dNTPs, 0.2 µM F3, 0.2 µM B3, 1.6 µM FIP, 1.6 µM BIP, 0.8 µM LF, 0.8 µM LB, REP (from 0.8 µM to 1.6 µM), 0.32 U µL^−1^ *Bst* 2.0 WarmStart DNA polymerase, RNase H2 (from 0.001 to 0.004 U µL^−1^), 0.1 mM HNB, 0.06 mM CR, 1 µL of the pUC57 plasmid targets, and nuclease‐free water. The real‐time fluorescence detection was conducted by incubating the reaction at 60 °C for 60 min in the Archimed X4 PCR instrument. For dual‐visualization assay, the color changes under ambient light were photographed using a smartphone and the dual‐color fluorescence changes under blue excitation were carried out in the portable blue LED transilluminator.

### Single‐Stage Mixed‐Dye‐Based REP‐LAMP Assays

The assay was achieved by directly adding WarmStart RTx into the mixed‐dye‐based REP‐LAMP reaction above. The optimized reaction contained 1× Low Buffer, 4 mM MgSO_4_, 1.6 mM each dNTPs, 0.2 µM F3, 0.2 µM B3, 1.6 µM FIP, 1.6 µM BIP, 0.8 µM LF, 0.8 µM LB, 1.2 µM REP, 0.32 U µL^−1^ *Bst* 2.0 WarmStart DNA polymerase, 0.002 U µl^−1^ RNase H2, 0.3 U µL^−1^ WarmStart RTx, 2.5 ng µL^−1^ ET SSB, 0.1 mM HNB and 0.06 mM CR, 1 µL of the transcribed RNA targets, and nuclease‐free water. Using the reaction system, the sensitivities on detecting various copies of transcribed RNA targets associated with Delta, BA2 and BA4/5 were tested through incubating the reaction at 60 °C for 60 min in the Archimed X4 PCR instrument.

### Dual‐Visualization Assays of REP‐TMAP

The REP‐TMAP assay was a closed‐tube, two‐stage assay in which the RT‐RPA mix with RNA targets placed on the tube's bottom initiates the first‐stage reaction and the mixed‐dye‐based REP‐LAMP mix placed on the tube's lid triggers the second‐stage reaction after brief spinning. Thus, a dual‐visualization REP‐TMAP reaction comprised the RT‐RPA mix and the mixed‐dye‐based REP‐LAMP mix. As a basic reaction, the RT‐RPA mix (10 µL) was prepared by mixing 0.1 U µL^−1^ RNase H, 2 U µL^−1^ Superscript IV RT, 0.2 µM F3, 0.2 µM B3, 14 mM MgOAc,1 µL of RNA targets, and 7.6 µL of the RPA mix which was formed by adding 38 µL rehydration buffer into a TwistAmp Basic reaction; the mixed‐dye‐based REP‐LAMP mix (10 µL) was prepared by mixing 1× Low Buffer, 4 mM MgSO_4_, 1.6 mM each dNTPs, 0.2 µM F3, 0.2 µM B3, 1.6 µM FIP, 1.6 µM BIP, 0.8 µM LF, 0.8 µM LB, 1.2 µM REP, 0.32 U µL^−1^ *Bst* 2.0 WarmStart DNA polymerase, 0.002 U µL^−1^ RNase H2, 0.1 mM HNB and 0.06 mM CR. Based on this basic reaction, the volume ratio of RT‐RPA mix to mixed‐dye‐based REP‐LAMP mix, the ratio of CR and HNB, and the volume of the mixed dyes were optimized. After obtaining the optimized reaction, the specificity and sensitivity of detecting various copies of transcribed RNA targets associated with JN.1, BA2, BA4/5, and Delta were tested. The REP‐TMAP reaction was first incubated for the first‐stage RT‐RPA by using a mini metal incubator at 42 °C for 20 min. Then, after fast centrifugation using a mini centrifuge, the reaction tube was placed in the Archimed X4 PCR instrument for the real‐time fluorescence REP‐LAMP assay (60 °C for 20 min). For dual‐visualization assay, the reaction tube was placed in the mini metal incubator at 60 °C for 20 min. The color changes under ambient light were photographed using a smartphone and the dual‐color fluorescence changes under blue excitation were conducted in the portable blue LED transilluminator.

### Clinical Sample Testing

The 102 human clinical oropharyngeal swabs were collected from volunteers and immediately placed into disposable virus sampling tubes which contained a previously reported viral inactivation and RNA preservation (VIP) buffer for simultaneous viral inactivation and RNA preservation.^[^ ^38^ ^]^ All the VIP buffer‐treated samples were kept at −20 °C until time of RNA extraction. The RNA extraction was performed using the QIAamp Viral RNA mini kit according to manufacture instructions in a Class II biosafety cabinet (NU545E‐300E, NuAire). The extracted RNA was stored at −80 °C before use. The dual‐visualization REP‐TMAP assays were conducted as described above, just replacing the targets with the RNA extracts. As parallel experiments, the commercial RT‐qPCR and the RT‐PCR plus Sanger sequencing were launched for confirming SARS‐CoV‐2 infection and for variant identification, respectively. The commercial RT‐qPCR targeted the ORF1ab, and N gene of SARS‐CoV‐2 and the reaction was conducted strictly following the kit's instructions. The RT‐PCR for sequencing was prepared using the HiScript II One Step RT‐PCR Kit and the amplified products were purified prior to Sanger sequencing. The RT‐PCR reaction (10 µL) contained 5 µL of 2× One Step Mix, 0.5 µL of One Step Enzyme Mix, 0.4 µM forward primer, 0.4 µM reverse primer, 1× EvaGreen, and 1 µL of RNA targets. The program of thermal cycling was 30 min at 50 °C for RT, 3 min at 94 °C for initial denaturation, 35 cycles of 30 s at 94 °C for denaturation, 30 s at 55 °C for annealing, and 1 min at 72 °C for extension, and 5 min at 72 °C for final extension. The RT‐PCR products were purified using the Universal DNA purification kit and the Sanger sequencing was finished by Sangon Biotech.

### Statistical Analysis

All statistical analyses including the confusion matrix of the test results were performed using GraphPad Prism 10.2.2 software. Statistical analysis was performed using the unpaired two‐tailed Student's *t*‐test (a *P* < 0.05 indicates statistical significance). Data were presented as mean ± standard deviation of at least three replicates. Unless otherwise specified, each image involving tube‐based visual detection in the corresponding figure is a representative of at least two independent experiments.

## Conflict of Interest

The authors declare no conflict of interest.

## Author Contributions

X.D. and Y.W. contributed equally to this work. X.D. designed methodology; performed validation and investigation; performed funding acquisition; wrote the original draft. Y.W. performed validation and investigation; wrote the original draft. Y.G. performed validation and investigation. C.Y. collected the clinical samples; performed validation and investigation.

## Supporting information

Supporting Information

## Data Availability

The data that support the findings of this study are available from the corresponding author upon reasonable request.
