# Supplementary material for: Two‐Stage Mixed‐Dye‐Based Isothermal Amplification with Ribonuclease‐Cleavable Enhanced Probes for Dual‐Visualization Detection of SARS‐CoV‐2 Variants of Interest
Source: Adv Sci (Weinh). 2024 Jun 3;11(29):2401988. doi: 10.1002/advs.202401988 (PMC11304323; doi:10.1002/advs.202401988)
Supplement: Supplementary file 1 — Supporting Information [file ADVS-11-2401988-s001.pdf]

ADVANCED SCIENCE
Open Access

Supporting Information

for *Adv. Sci.*, DOI 10.1002/advs.202401988

Two-Stage Mixed-Dye-Based Isothermal Amplification with Ribonuclease-Cleavable Enhanced Probes for Dual-Visualization Detection of SARS-CoV-2 Variants of Interest

Xiong Ding, Yaru Wang, Yuxin Gui and Chuankun Yang*

Supporting Information

Two-stage Mixed-dye-based Isothermal Amplification with Ribonuclease-cleavable Enhanced Probes for Dual-visualization Detection of SARS-CoV-2 Variants of Interest

Xiong Ding, Yaru Wang, Yuxing Gui, and Chuankun Yang*

Xiong Ding, Yaru Wang, Yuxing Gui
Key Laboratory of Environmental Medicine and Engineering, Ministry of Education
Department of Nutrition and Food Hygiene, School of Public Health
Southeast University
Nanjing 210009, P. R. China
E-mail: xiongdlab21@seu.edu.cn

Chuankun Yang
Center of Clinical Laboratory Medicine
Zhongda Hospital
Southeast University
Nanjing 210009, P. R. China

Xiong Ding and Yaru Wang contributed equally to this work

Table of Contents

Figure S1. Scheme of REP-LAMP assay for the detection of mutant-type (MT) target sequence with a single base mutation........ **3**
Figure S2. Effect of various sizes of REP's extensions at ends on REP-LAMP assays........... **4**
Figure S3. REP-LAMP assays with or without LF primer and the REP residue analysis **5**
Figure S4. Free energy of the secondary structure of the used CBP sequence predicted by the software NUPACK (Caltech) **6**
Figure S5. Visual LAMP assays by using A) CR, (B) HNB, and C) the mixed dyes of HNB and CR **7**
Figure S6. Effect of various concentrations of A) REP and B) RNase H2 on dual-visualization assays by mixed-dye-based REP-LAMP........ **7**

Figure S7. Single-stage mixed-dye-based REP-LAMP assays. A) Workflow diagram of single-stage mixed-dye-based REP-LAMP **8**
Figure S8. Real-time fluorescence detection by single-stage mixed-dye-based REP-LAMP.. **9**
Figure S9. Effect of various volumes of the mixed dyes on real-time fluorescence mutation detection by REP-TMAP **10**
Figure S10. Real-time fluorescence REP-TMAP assays **11**
Figure S11. Specificities of the real-time fluorescence REP-TMAP assays for A) JN.1, B) BA.4/5, C) BA.2, and D) Delta S gene RNA targets **12**
Figure S12. Sample testing using dual-visualization REP-TMAP assays **13**
Figure S13. Sample testing using the commercial RT-qPCR assays. **16**
Table S1. The results of Sanger sequencing for variant identification of the clinical samples with SARS-CoV-2 positive **20**
Table S2. The cycle quantification (Cq) values of the 102 samples by the commercial RT-qPCR targeting ORF1ab, N, and POP7 genes **33**
Table S3. Comparison of isothermal amplification strategies for the detection of SARS-CoV-2 nucleic acids........ **35**
Table S4. The estimated cost of the REP-TMAP assay........ **37**
Table S5. The list of all used plasmids in this study........ **38**
Table S6. The list of all used primers and probes in this study **43**
References **46**

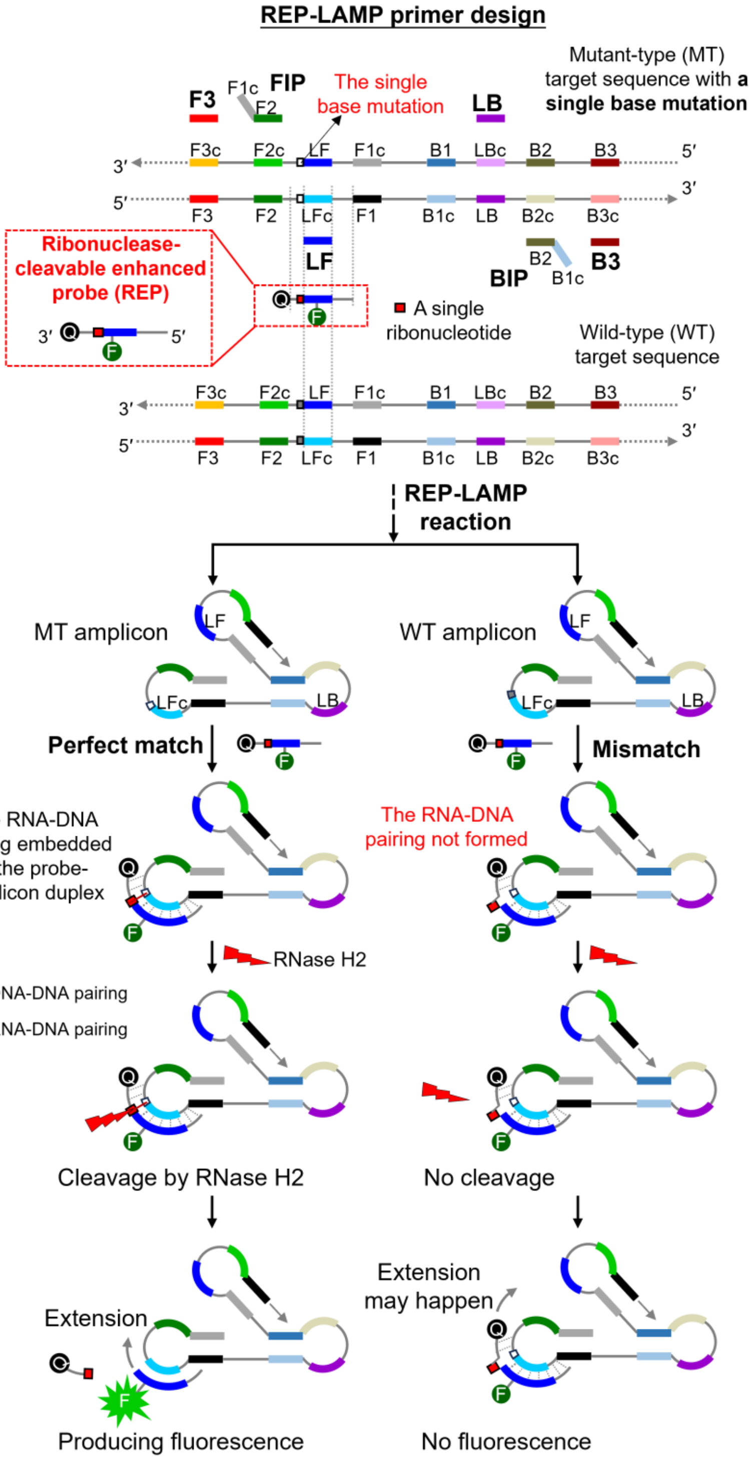

Figure S1. Scheme of REP-LAMP assay for the detection of mutant-type (MT) target sequence with a single base mutation.

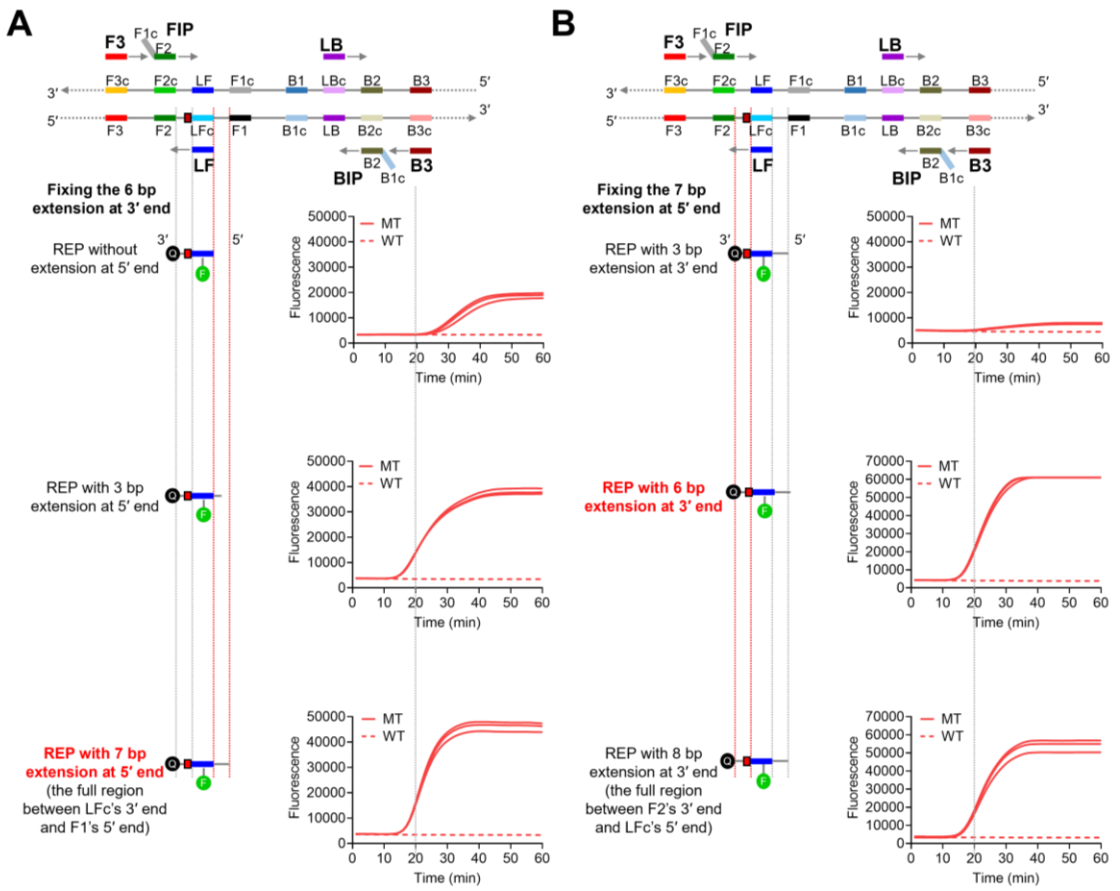

Figure S2. Effect of various sizes of REP's extensions at ends on REP-LAMP assays. A) Real-time fluorescence detection of REP-LAMP with various sizes of extensions at 5′ end when fixing the extensions at 3′ end as 6 bp. MT, the positive reactions with 10^5 copies of the pUC57 plasmids containing the S gene fragment with the mutation of DEL21633-21640 (5′-TACCCCCT-3′). WT, the positive reactions with 10^5 copies of the pUC57 plasmids containing the wild-type (WT) S gene fragment. B) Real-time fluorescence detection of REP-LAMP with various sizes of extensions at 3′ end when fixing the extensions at 5′ end as 7 bp. MT, the positive reactions with 2×10^5 copies of the pUC57 plasmids containing the mutated S gene fragment. WT, the positive reactions with 2×10^5 copies of the pUC57 plasmids containing WT S gene fragment. Three replicates (n=3) were run for each MT test.

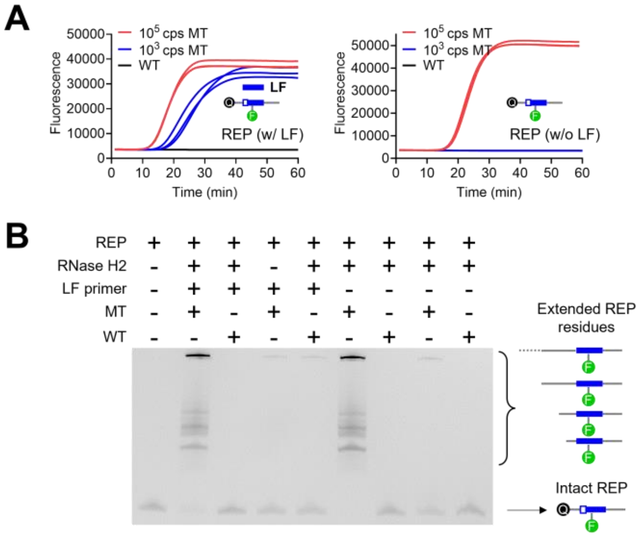

Figure S3. REP-LAMP assays with or without LF primer and the REP residue analysis. A) Effect of LF primer on REP-LAMP assay with 10^5 and 10^3 copies MT targets. B) Denaturing PAGE analysis of amplified REP residues after RNase H2 cleavage in REP-LAMP assays with and without the LF primer. MT, the positive reactions with 10^5 and 10^3 copies of the pUC57 plasmids containing the S gene fragment with the mutation of DEL21633-21640 (5′-TACCCCCT-3′). WT, the positive reactions with 10^5 copies of the pUC57 plasmids containing the wild-type (WT) S gene fragment. Three replicates (n=3) were run for each MT test.

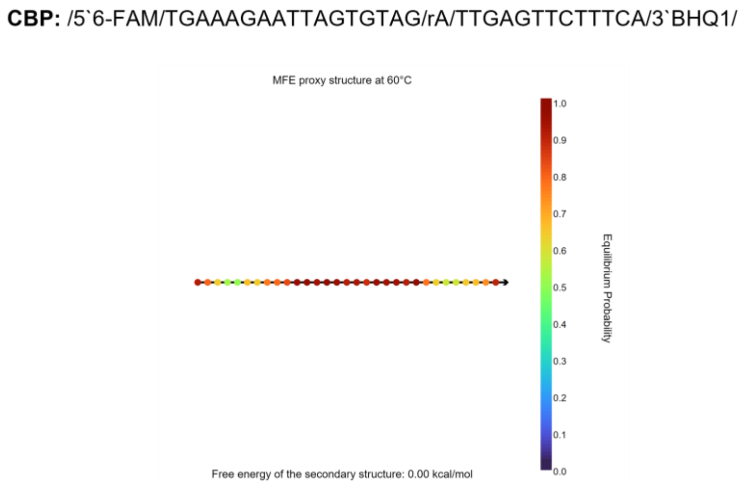

Figure S4. Free energy of the secondary structure of the used CBP sequence predicted by the software NUPACK (Caltech). The CBP sequence was shown in Table S5 and the conditions for prediction were 0.8 μM CBP, 70 mM Na^+, and 6 mM Mg^{2+} at 60°C.

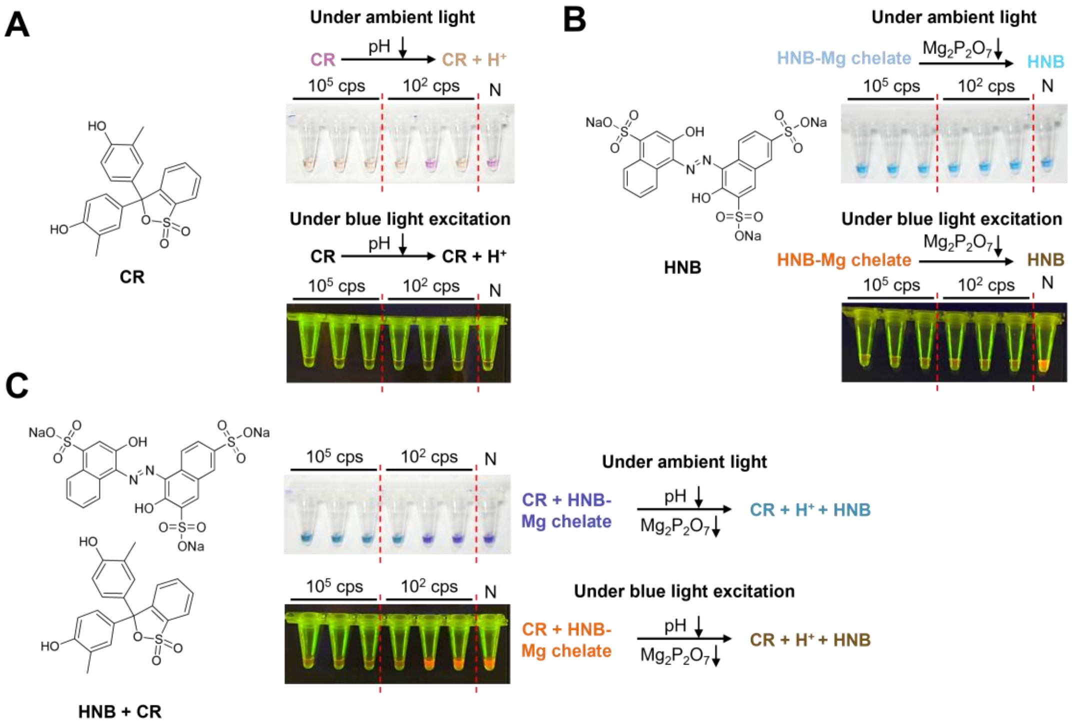

Figure S5. Visual LAMP assays by using A) CR, (B) HNB, and C) the mixed dyes of HNB and CR. P, the positive LAMP reactions with 10^5 or 10^2 copies of the pUC57 plasmids containing the MT S gene fragment of DEL21633-21640 (5′-TACCCCCT-3′). N, the negative LAMP reactions without the targets. Each image of tube-based visual detection is a representative of three independent experiments. Three replicates (n=3) were run for each positive test.

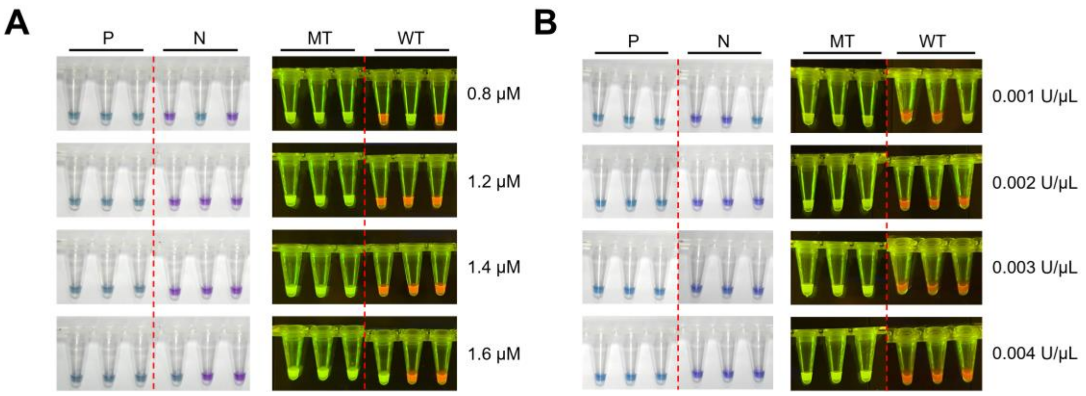

Figure S6. Effect of various concentrations of A) REP and B) RNase H2 on dual-visualization assays by mixed-dye-based REP-LAMP. P and MT, the positive reactions with 10^5 copies of the pUC57 plasmids containing the MT S gene fragment of DEL21633-21640 (5′-TACCCCCT-3′). N, the negative reactions without the targets. WT, the reactions with 10^5 copies of the pUC57 plasmids containing WT S gene fragment. Each image of tube-based visual detection is a representative of three independent experiments. Three replicates (n=3) were run for each test.

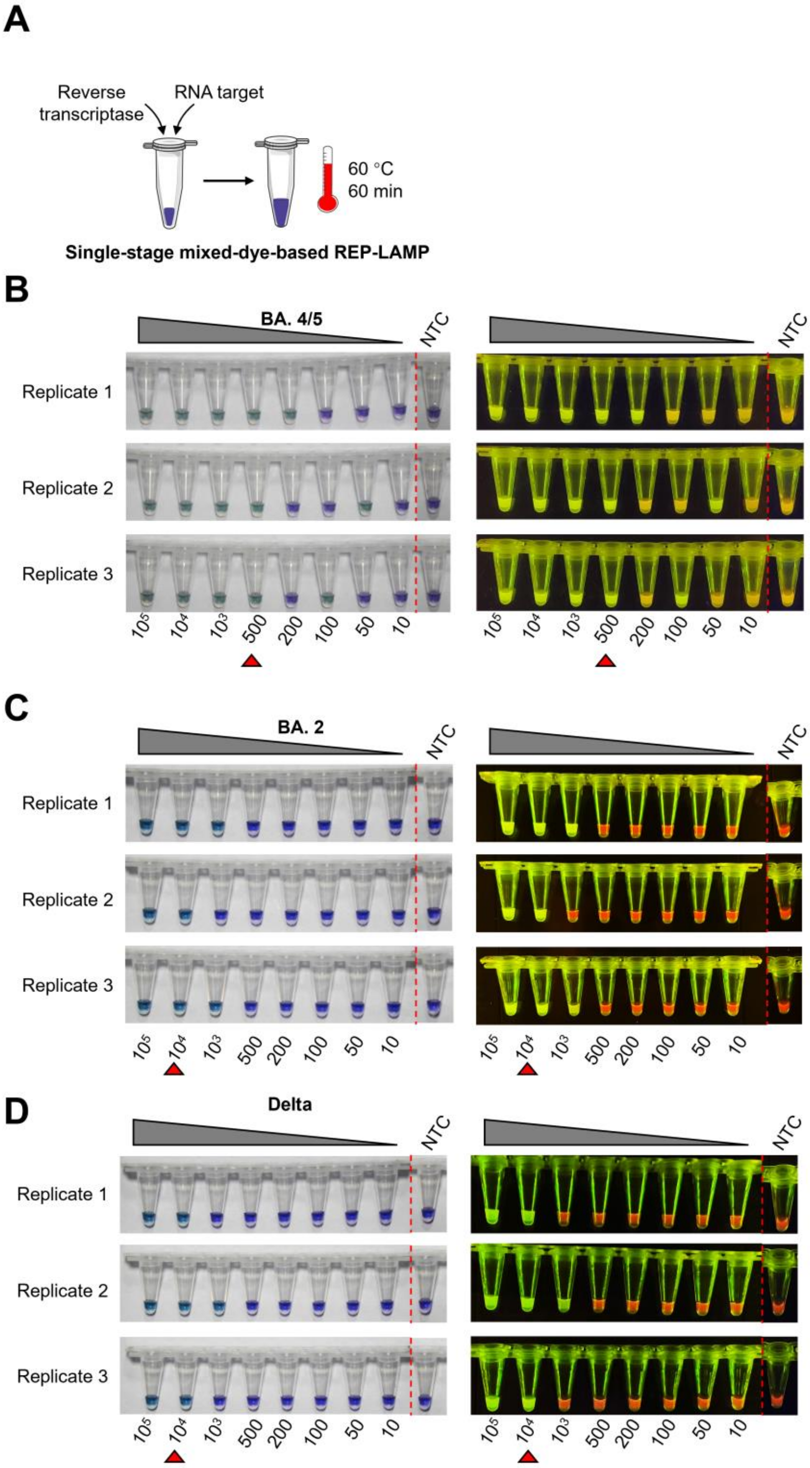

Figure S7. Single-stage mixed-dye-based REP-LAMP assays. A) Workflow diagram of single-stage mixed-dye-based REP-LAMP. B-D) Sensitivities of dual-visualization assays of mixed-dye-based REP-LAMP on detecting various copies of S gene RNA targets associated with BA.4/5, BA.2, and Delta variants, respectively. NTC, no-template control. Three replicates (n=3) were run for each test.

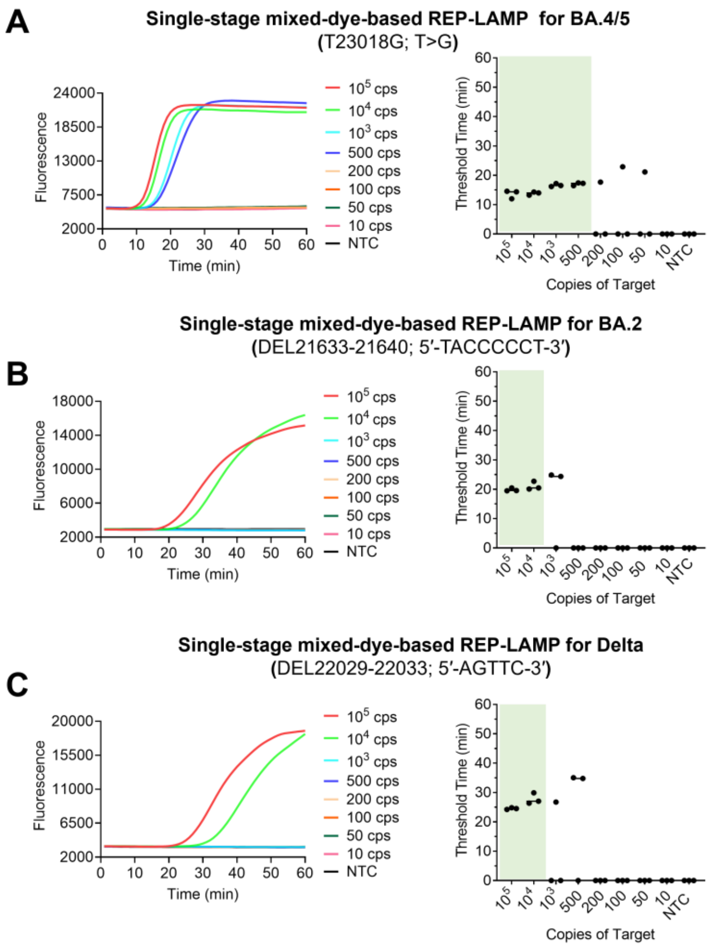

Figure S8. Real-time fluorescence detection by single-stage mixed-dye-based REP-LAMP. A-C) Sensitivities and threshold time comparison of real-time fluorescence assays on detecting various copies (cps) of S gene RNA targets associated with BA.4/5, BA.2, and Delta variants, respectively. NTC, no-template control. Three replicates were run for each test.

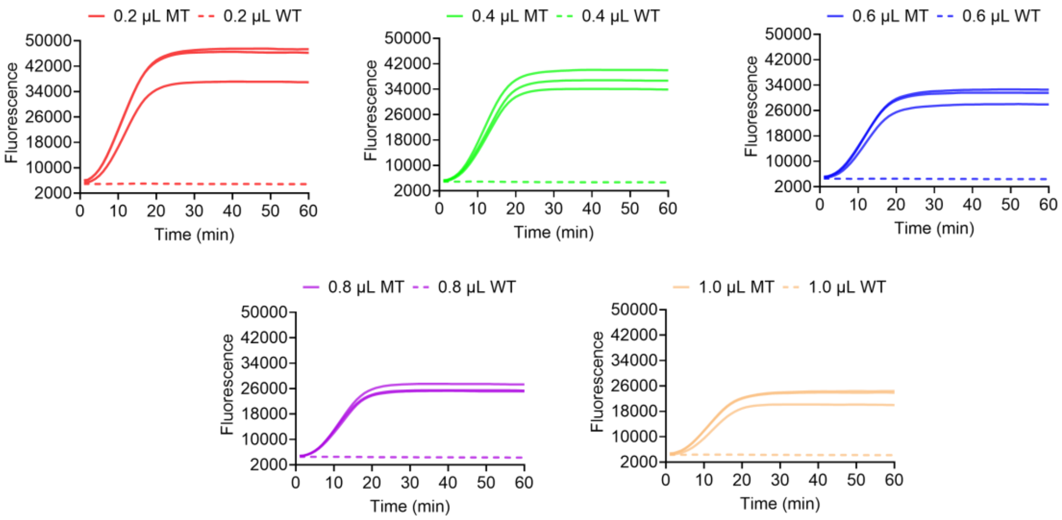

Figure S9. Effect of various volumes of the mixed dyes on real-time fluorescence mutation detection by REP-TMAP. Only the green fluorescence from FAM fluorophore is real-timely recorded. MT, the positive reactions with 10^5 copies of transcribed RNA targets containing the MT S gene fragment of DEL21633-21640 (5′-TACCCCCT-3′). N, the negative reactions without the targets. WT, the reactions with 10^5 copies of transcribed RNA targets containing WT S gene fragment. Three replicates (n=3) were run for each positive test.

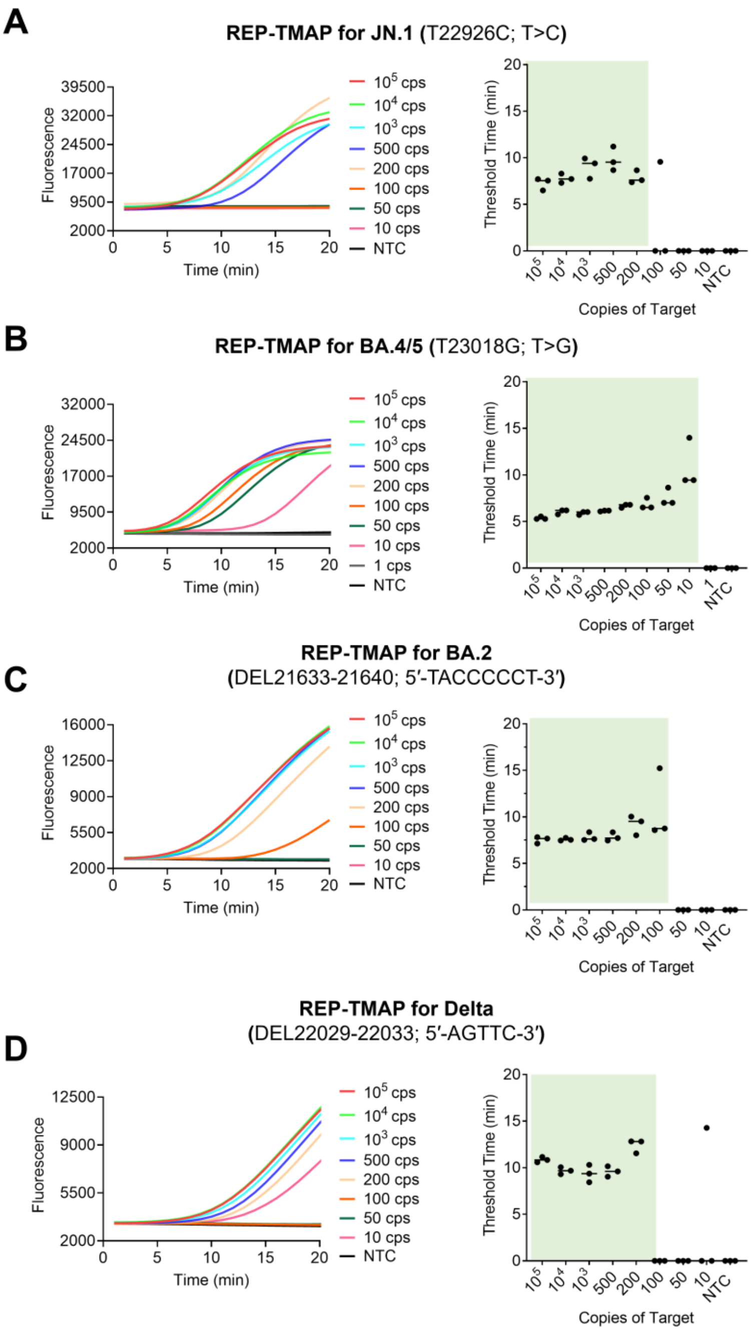

Figure S10. Real-time fluorescence REP-TMAP assays. A-D) Sensitivities and threshold time comparison of real-time fluorescence REP-TMAP assays on detecting various copies (cps) of S gene RNA targets associated with JN.1, BA.4/5, BA.2, and Delta variants, respectively. Only the green fluorescence from FAM fluorophore is real-timely recorded. NTC, no-template control. Three replicates (n=3) were run for each test.

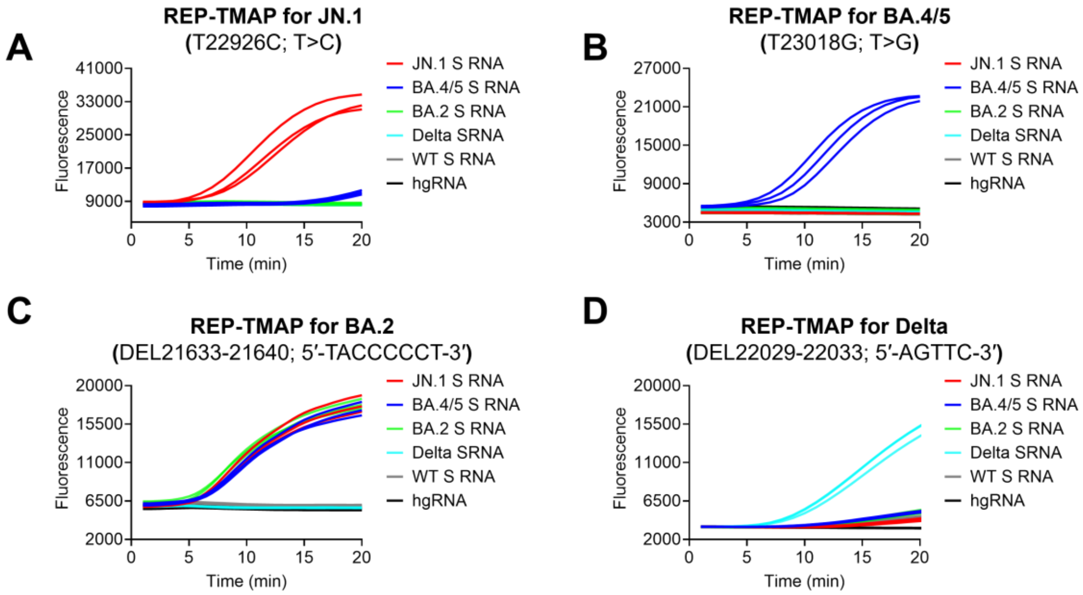

Figure S11. Specificities of the real-time fluorescence REP-TMAP assays for A) JN.1, B) BA.4/5, C) BA.2, and D) Delta S gene RNA targets. Only the green fluorescence from FAM fluorophore is real-timely recorded. hgRNA, human genomic RNA. All the targets with 10^4 copies (cps) were loaded into each reaction. Three replicates (n=3) were run for each test.

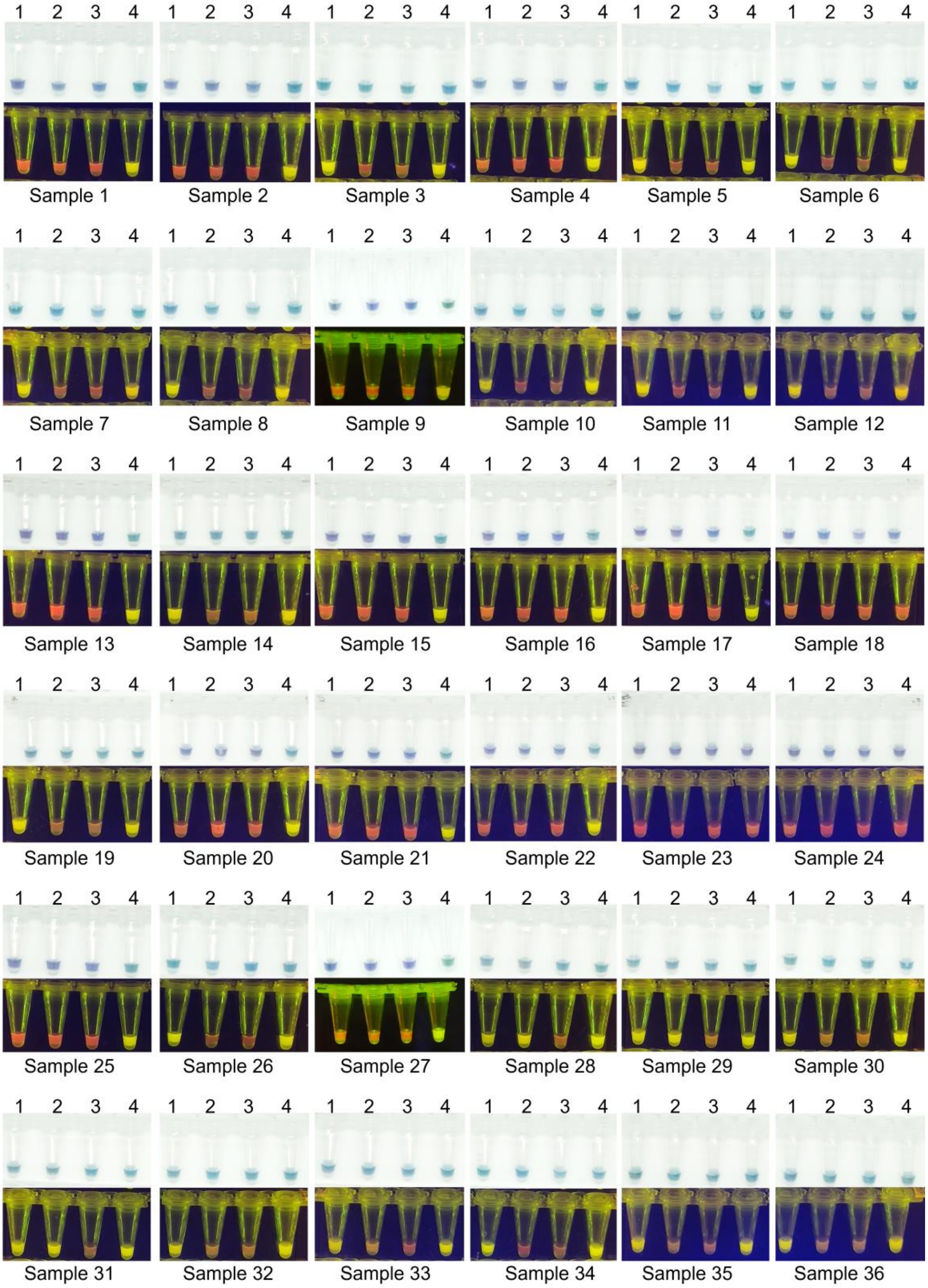

Figure S12. Sample testing using dual-visualization REP-TMAP assays. Tube 1-4, the identification of DEL21633-21640 (5′-TACCCCCT-3′) for BA.2 S protein mutation DEL25/27, T23018G (T>G) for BA.4/5 S protein mutation F486V, T22926C (T>C) for JN.1 S protein mutation L455S, and the POP7 control gene.

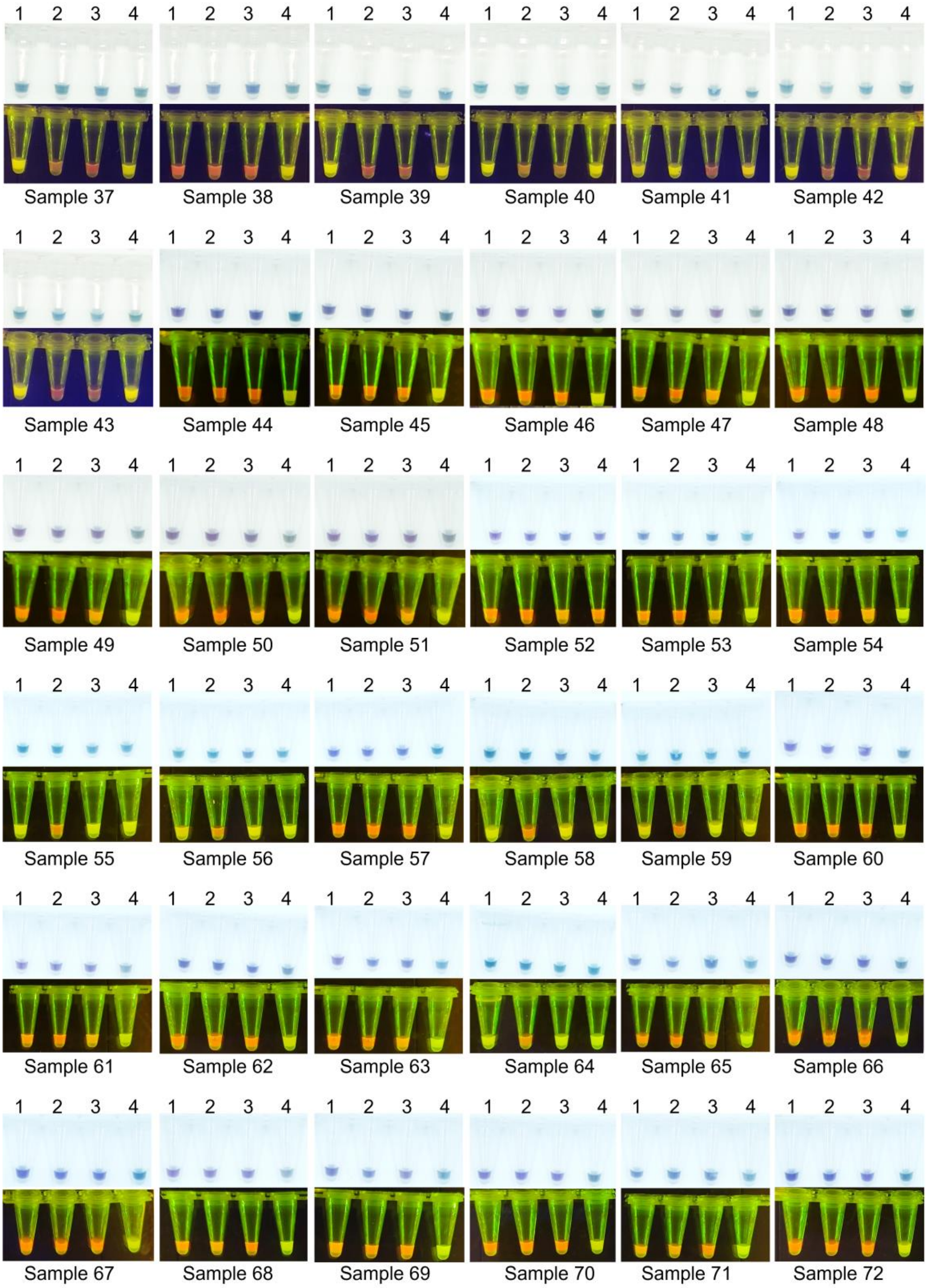

Figure S12 (continued). **Sample testing using dual-visualization REP-TMAP assays.** Tube 1-4, the identification of DEL21633-21640 (5′-TACCCCCT-3′) for BA.2 S protein mutation DEL25/27, T23018G (T>G) for BA.4/5 S protein mutation F486V, T22926C (T>C) for JN.1 S protein mutation L455S, and the POP7 control gene.

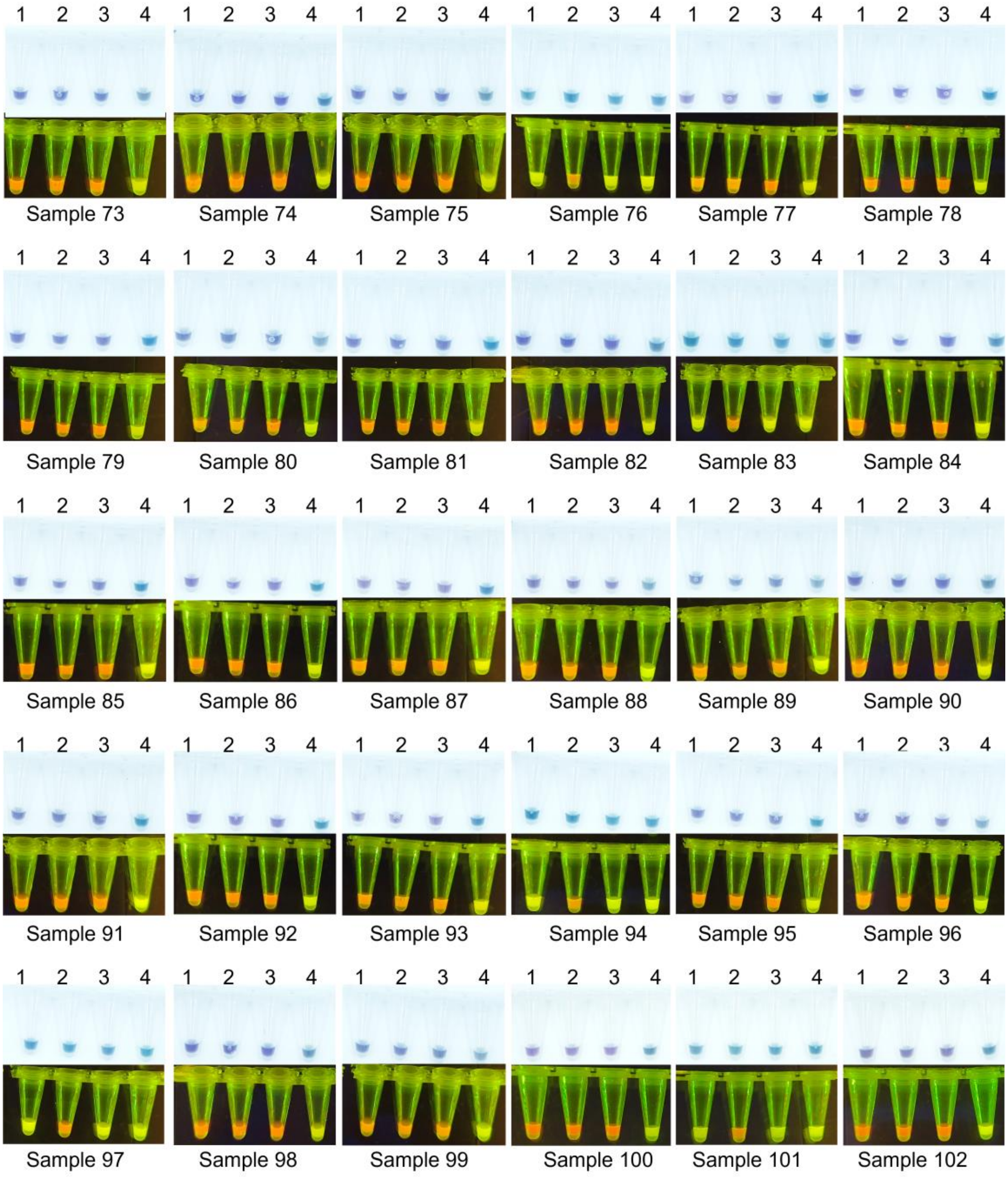

Figure S12 (continued). **Sample testing using dual-visualization REP-TMAP assays.** Tube 1-4, the identification of DEL21633-21640 (5′-TACCCCCT-3′) for BA.2 S protein mutation DEL25/27, T23018G (T>G) for BA.4/5 S protein mutation F486V, T22926C (T>C) for JN.1 S protein mutation L455S, and the POP7 control gene.

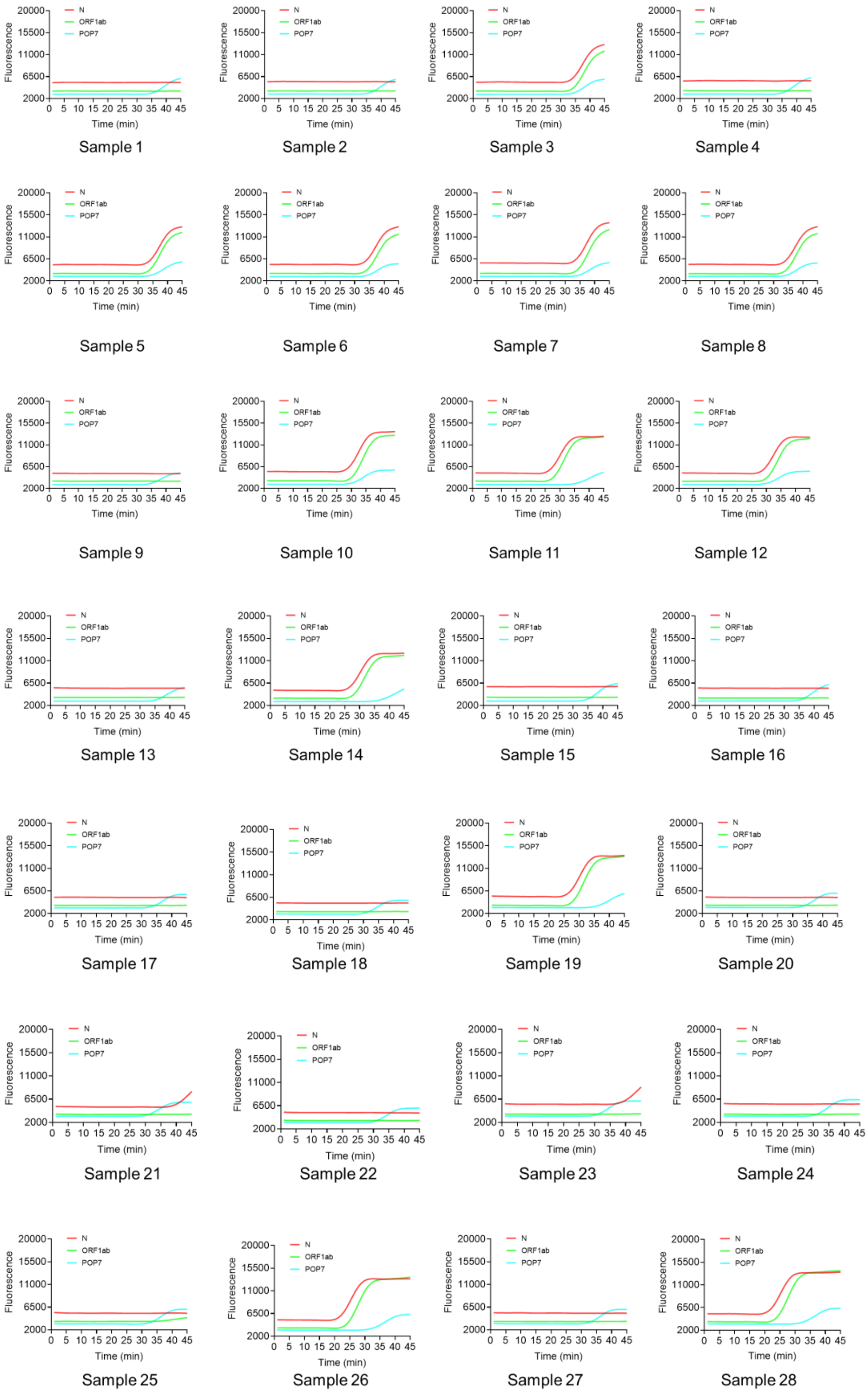

Figure S13. Sample testing using the commercial RT-qPCR assays.

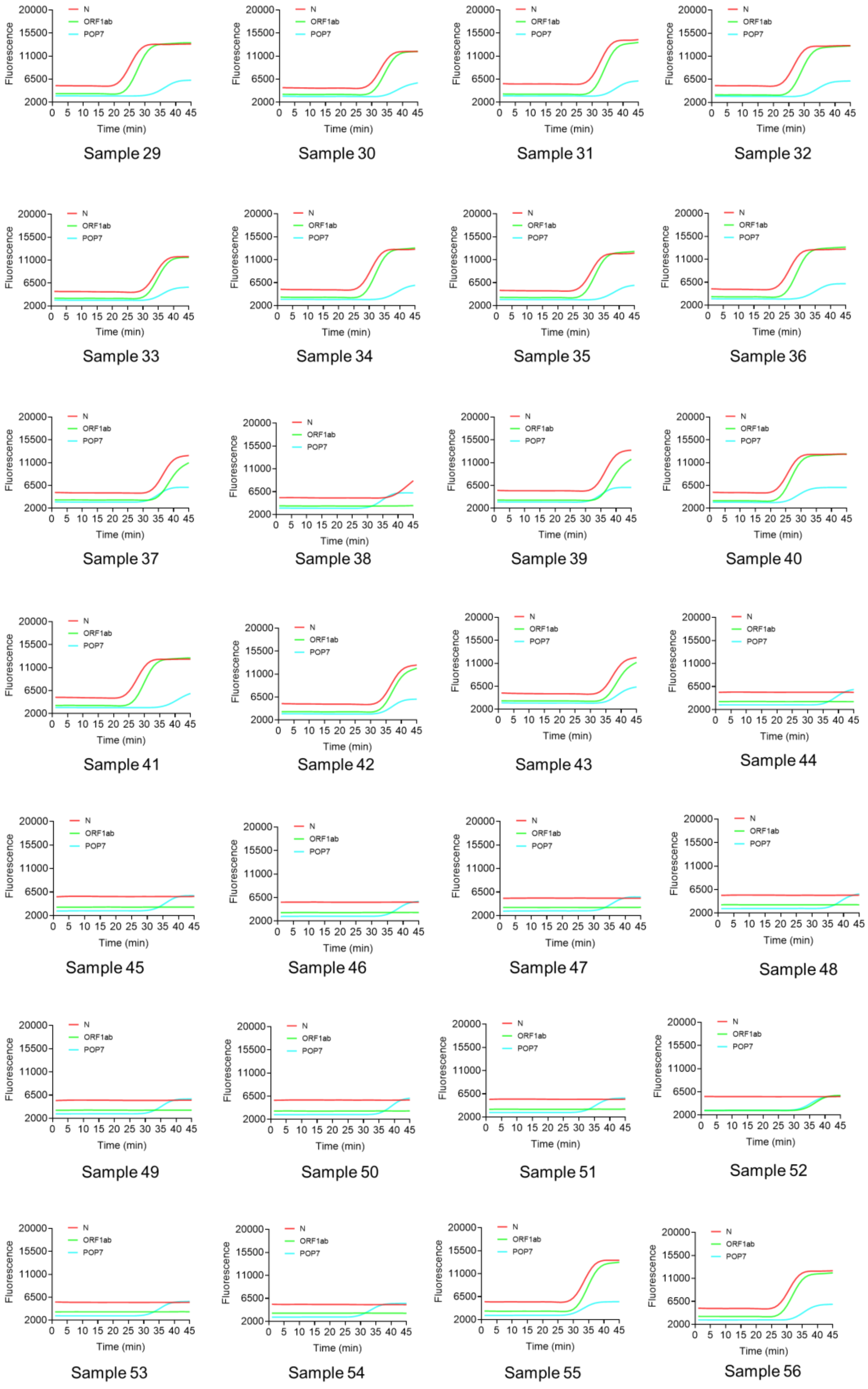

Figure S13 (continued). Sample testing using the commercial RT-qPCR assays.

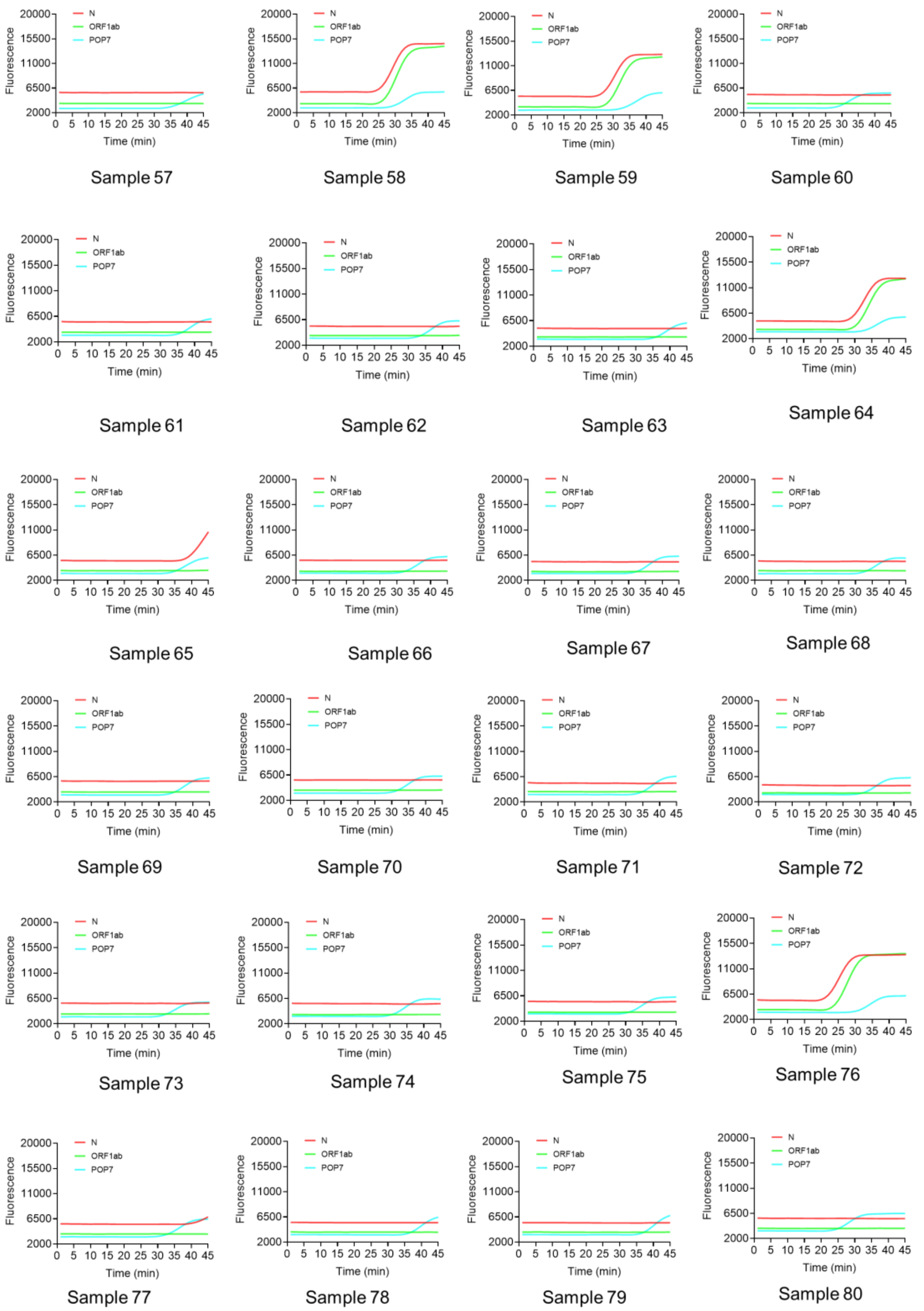

Figure S13 (continued). Sample testing using the commercial RT-qPCR assays.

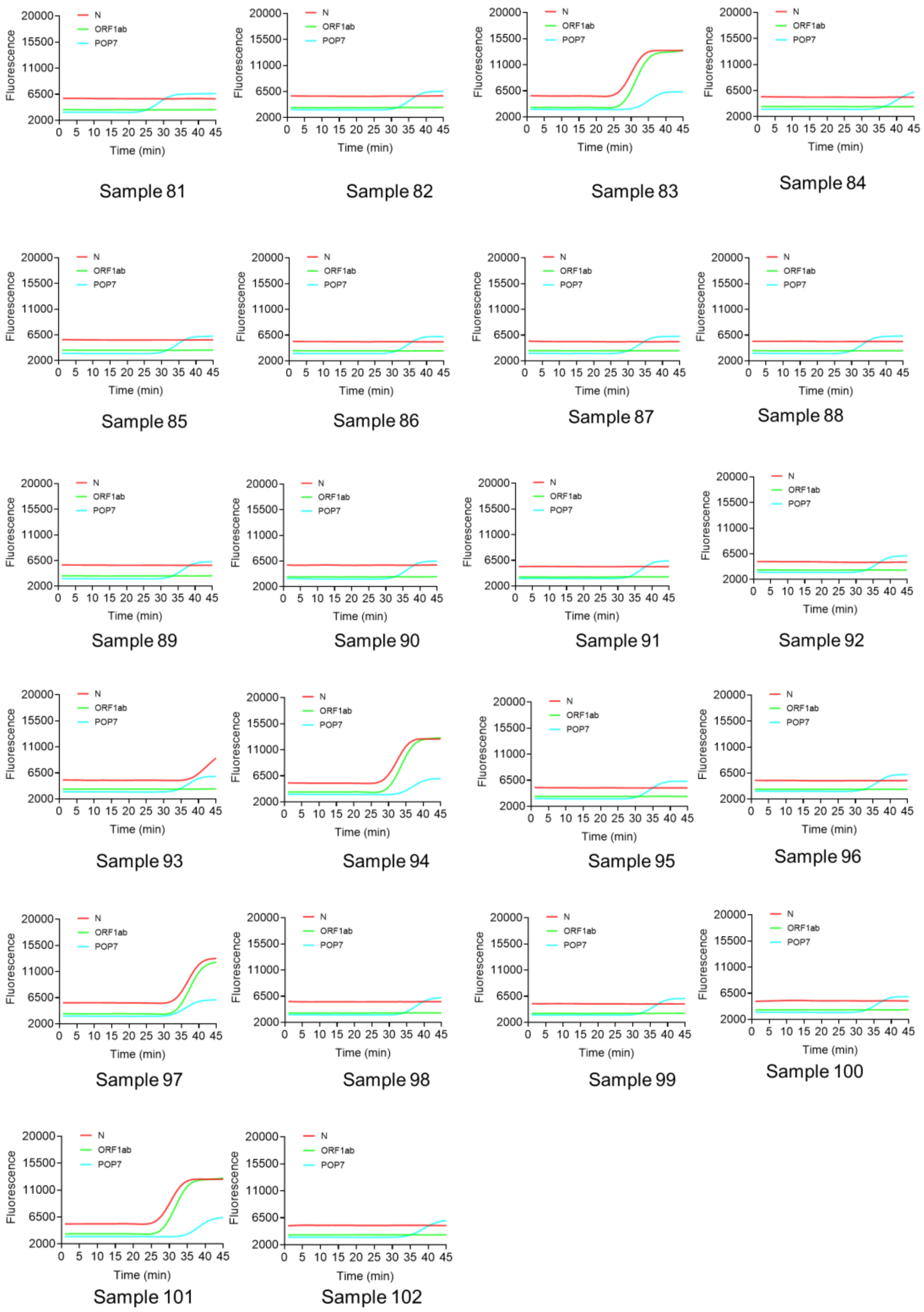

Figure S13 (continued). Sample testing using the commercial RT-qPCR assays.

Table S1. The results of Sanger sequencing for variant identification of the clinical samples with SARS-CoV-2 positive.

Sample 3	AGAACTCAT--------CATACACTAATTCTTTCACACGTGGTGTTT 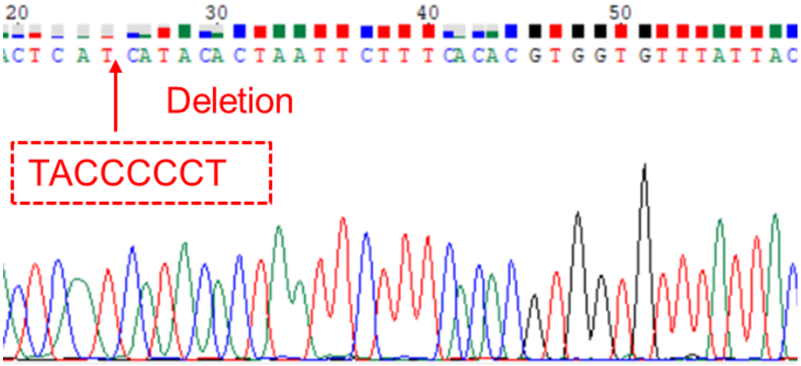
Sample 5	AGAACTCAT--------CATACACTAATTCTTTCACACGTGGTGTTT 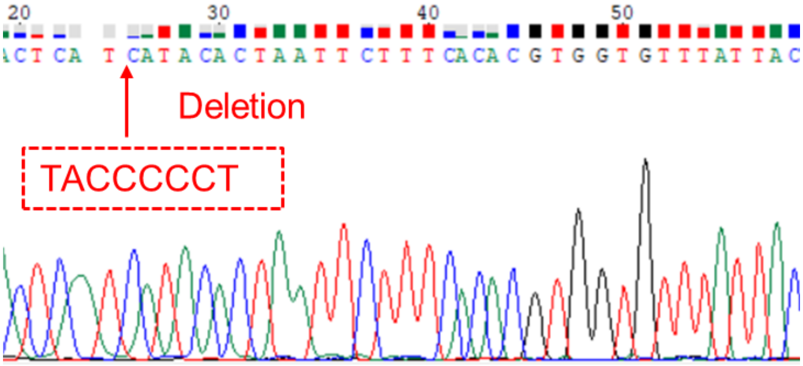
Sample 6	AGAACTCAT--------CATACACTAATTCTTTCACACGTGGTGTTT 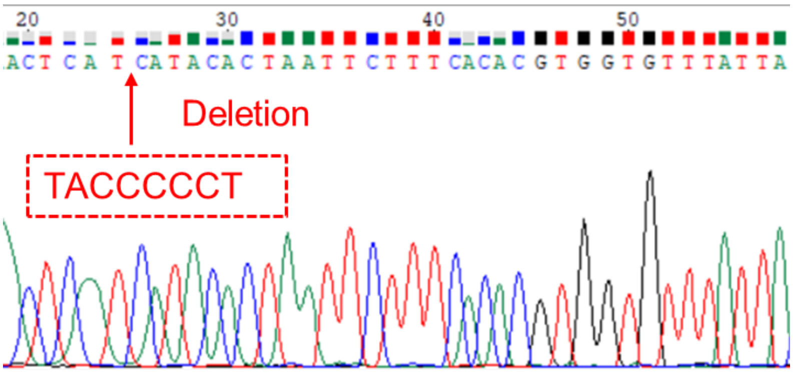
Sample 7	AGAACTCAT--------CATACACTAATTCTTTCACACGTGGTGTTT 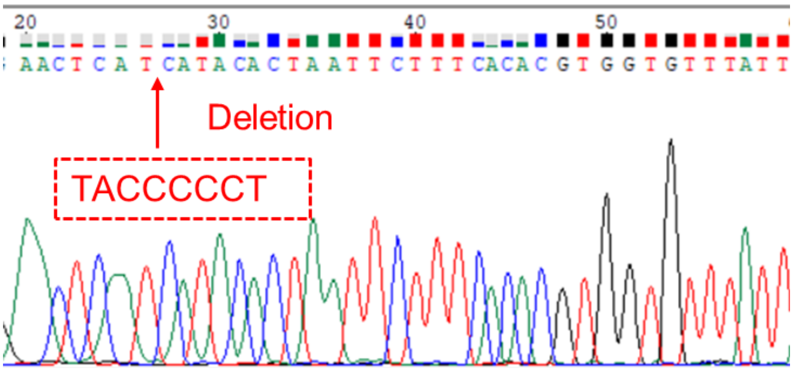
Sample 8	AGAACTCAT--------CATACACTAATTCTTTCACACGTGGTGTTT

	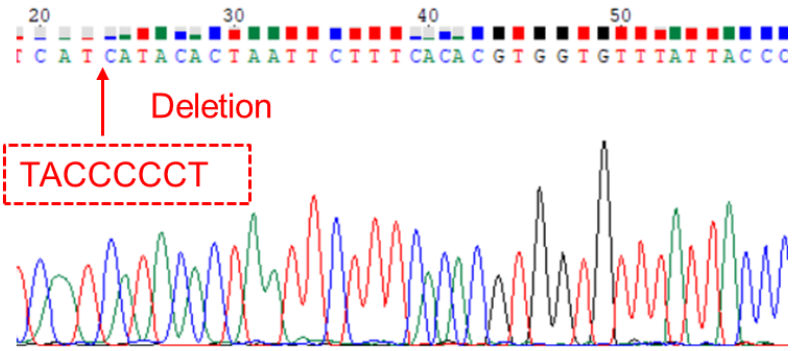
Sample 10	AGACTCAT--------CATACACTAGATTCTTTCCACCTGGTGTTT<br>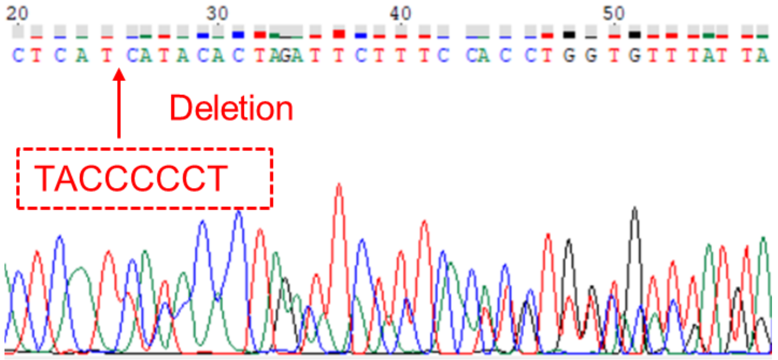
Sample 11	AGAACTCAT--------CATACACTAGATTCTTTCACACGTGGTGTTT<br>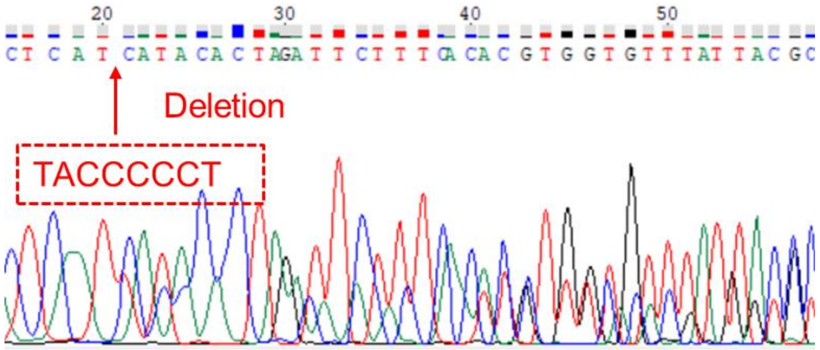
Sample 12	AGAACTCAT--------CATACACTAATTCTTTCCACGTGGTGTTT<br>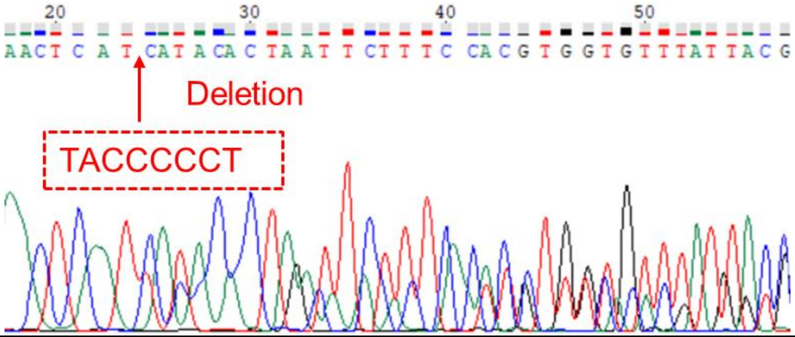
Sample 14	AGAACTCAT--------CATACACTAATTCTTTCACACGTGGTGTTT

<table>
<tr><td></td><td>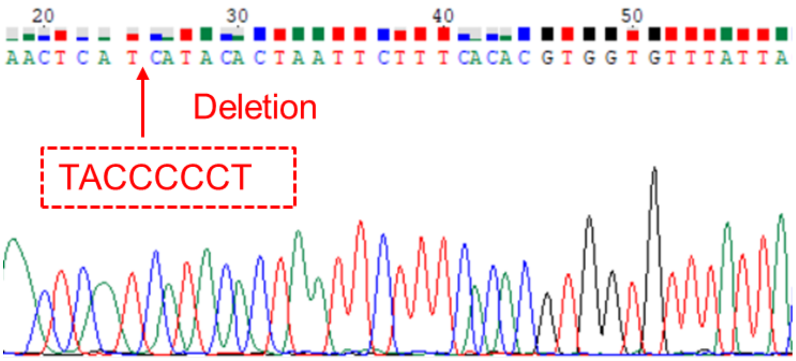
</td></tr>
<tr><td>Sample 19</td><td>AGACTCAA--------CATACACTAATTCTTTCACACGTGGTGTTT
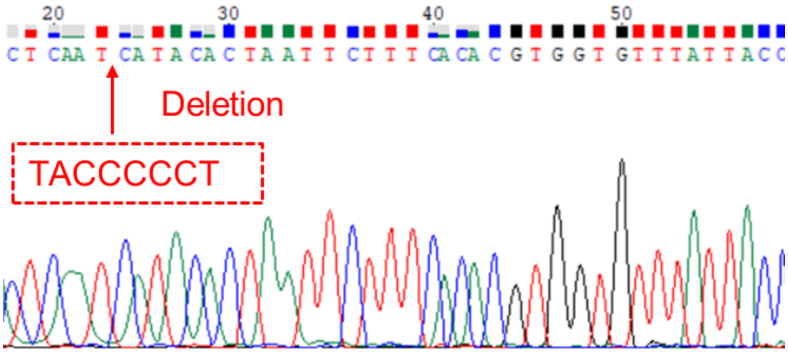
</td></tr>
<tr><td>Sample 26</td><td>AGGACTCAAT--------CATACACTAATTCTTTCACACGTGGTGTTT
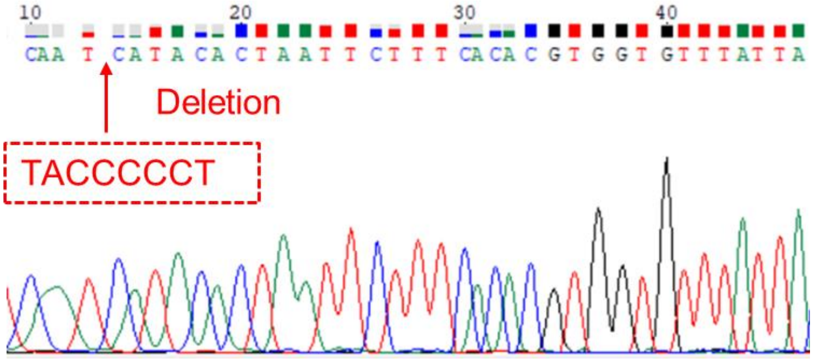
</td></tr>
<tr><td>Sample 28</td><td>AGAACTCAAT--------CATACACTAATTCTTTCACACGTGGTGTTT
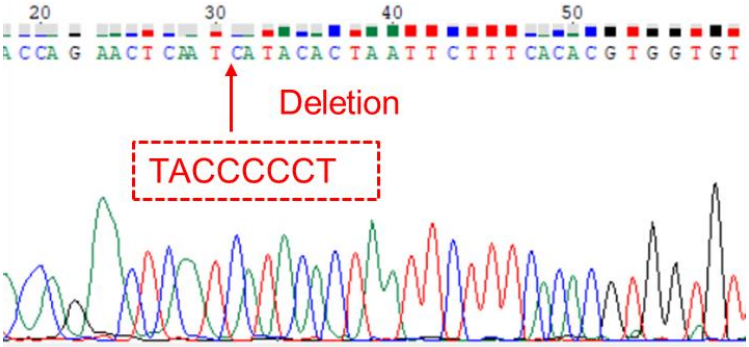

TAATGGTGTTGCAGGTGTTAATTGTTACTTTCCTTTACA</td></tr>
</table>

<table>
<tr><td></td><td>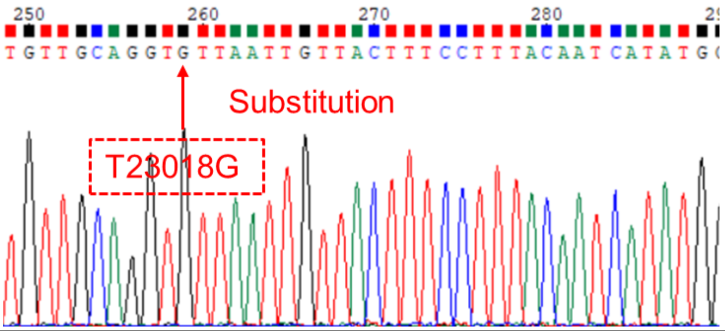
</td></tr>
<tr><td>Sample 29</td><td>AGACTCAT--------CATACACTAATTCTTTCCACCTGGTGTTT
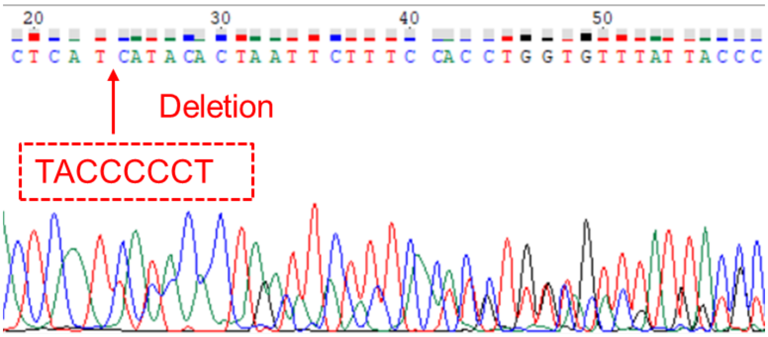

TAATGGTGTTGCAGGTGTTAATTGTTACTTTCCTTTACA
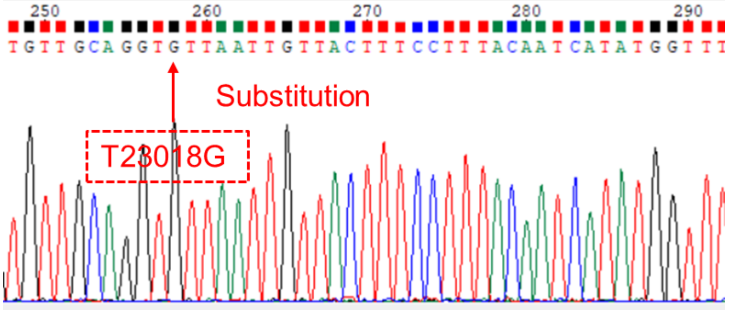
</td></tr>
<tr><td>Sample 30</td><td>AGAACTCAAT--------CATACACTAATTCTTTCACACGTGGTGTTT
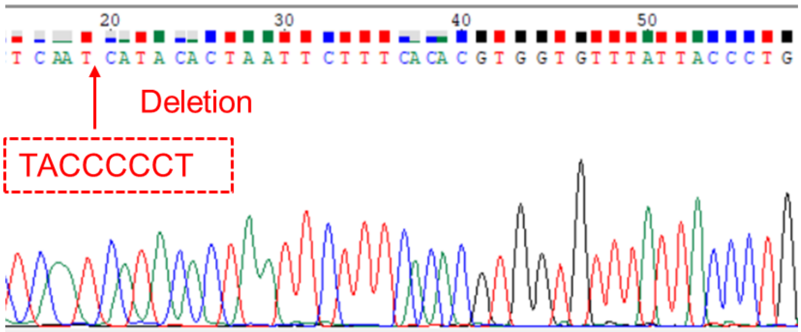
</td></tr>
<tr><td>Sample 31</td><td>AGAACTCAAT--------CATACACTAATTCTTTCACACGTGGTGTTT</td></tr>
</table>

	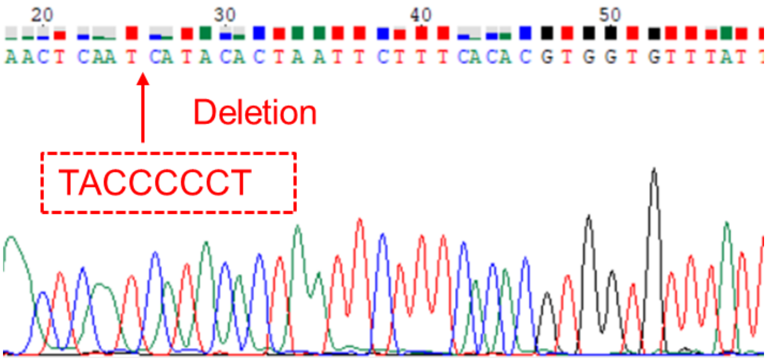<br>TAATGGTGTTGCAGGTGTTAATTGTTATTTTCCTTTACA<br>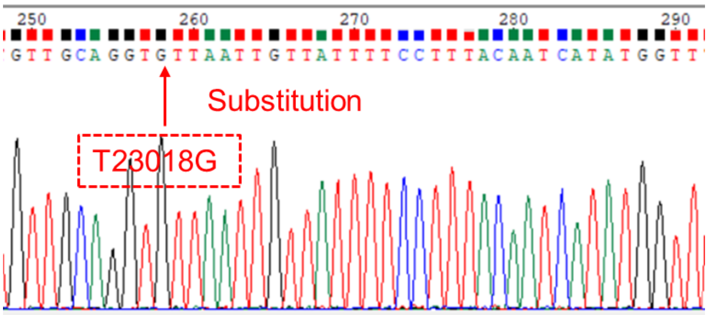
Sample 32	AGACTCAT--------CATACACTAATTCTTTCACACGTGGTGTTT<br>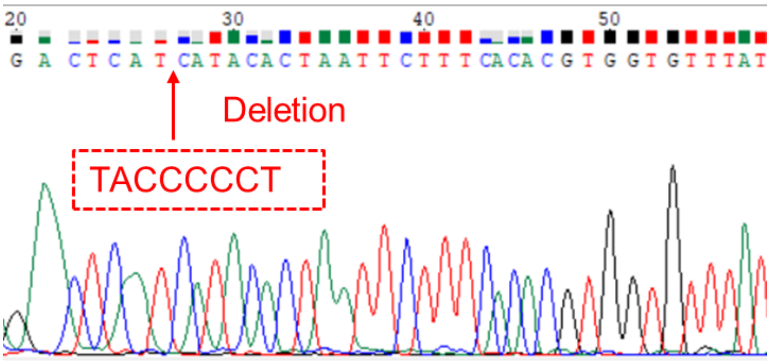
Sample 33	AGAACTCAAT--------CATACACTAATTCTTTCACACGTGGTGTTT<br>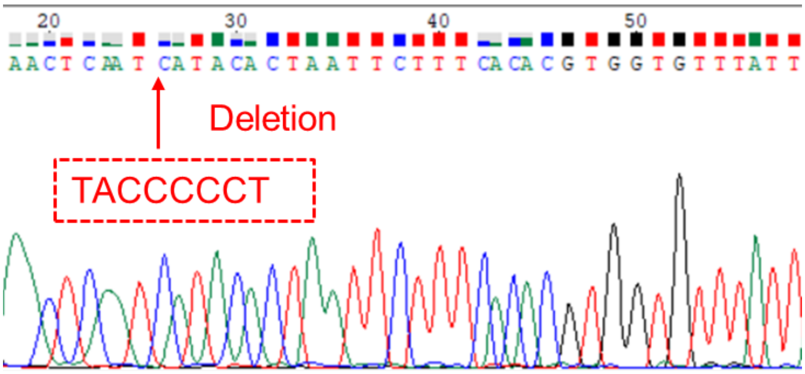
Sample 34	AGAACTCAAT--------CATACACTAATTCTTTCACACGTGGTGTTT

	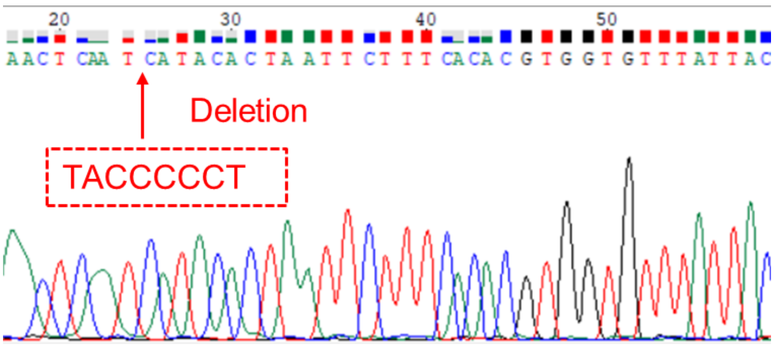
Sample 35	AGACTCAAT--------CATACACTAATTCTTTCACACGTGGTGTTT 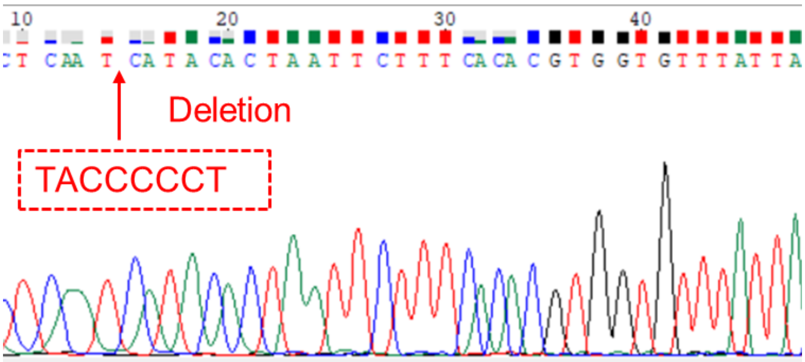
Sample 36	AGAACTCAAT--------CATACACTAATTCTTTCACACGTGGTGTTT 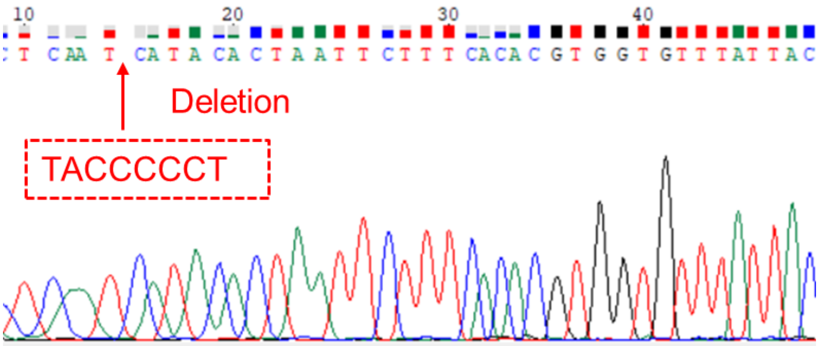
Sample 37	AGAACTCAAT--------CATACACTAATTCTTTCACACGTGGTGTTT 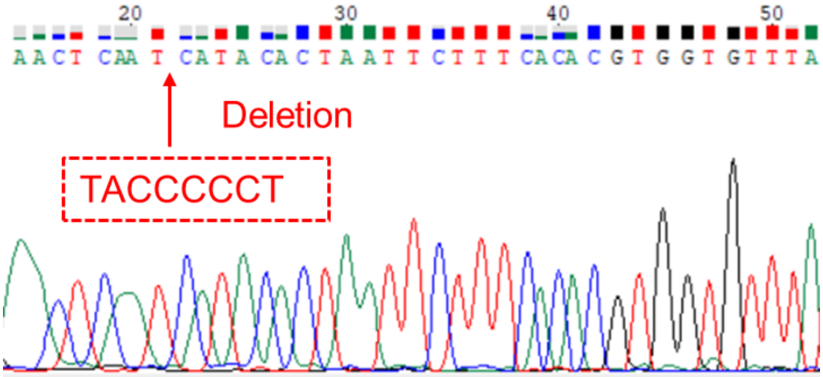
Sample 39	AGAACTCAAT--------CATACACTAATTCTTTCACACGTGGTGTTT

<table>
<tr><td></td><td>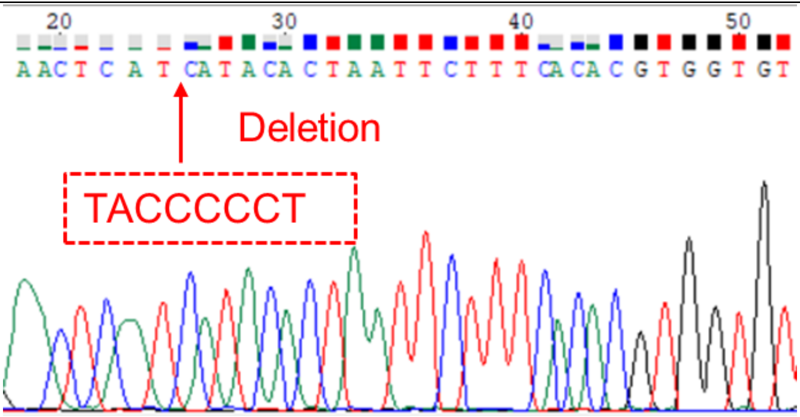
</td></tr>
<tr><td>Sample 40</td><td>AGACTCAAT--------CATACACTAATTCTTTCACACGTGGTGTTT
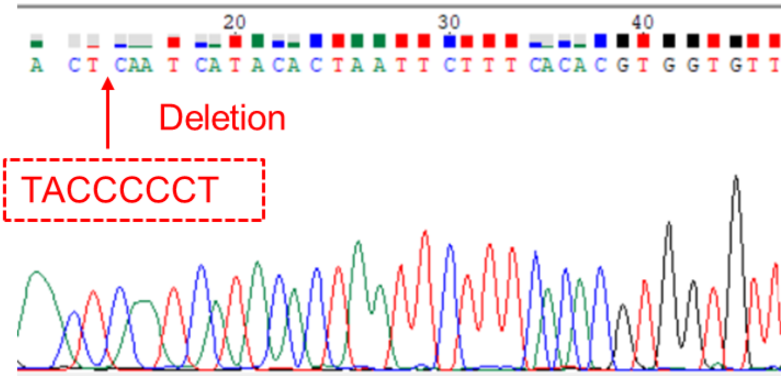
</td></tr>
<tr><td>Sample 41</td><td>AGACTCAAT--------CATACACTAATTCTTTCACACGTGGTGTTT
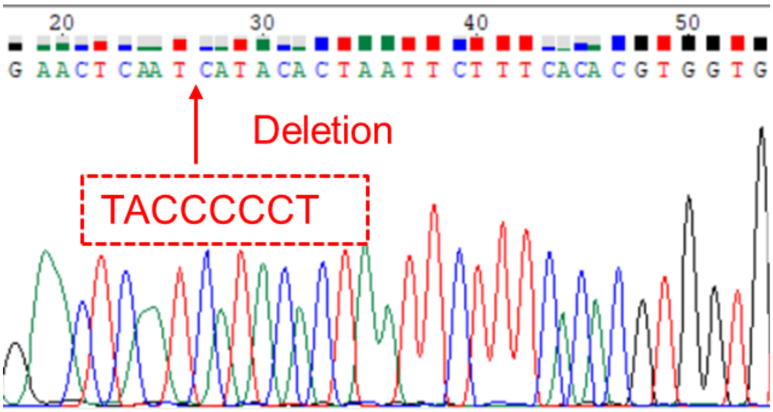

TAATGGTGTTGCAGGTGTTAATTGTTACTTTCCTTTACA
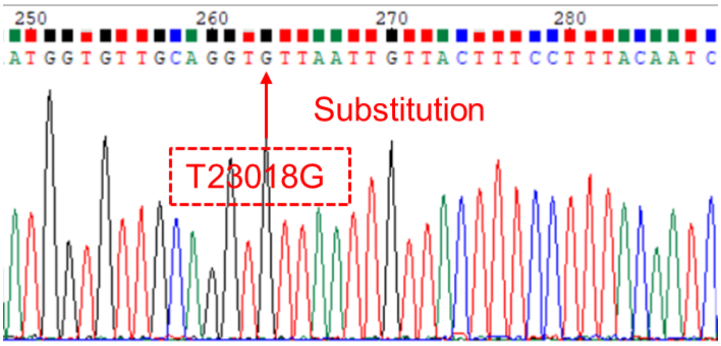
</td></tr>
<tr><td>Sample 42</td><td>AGAACTCAAT--------CATACACTAATTCTTTCACACGTGGTGTTT</td></tr>
</table>

<table>
<tr><td></td><td>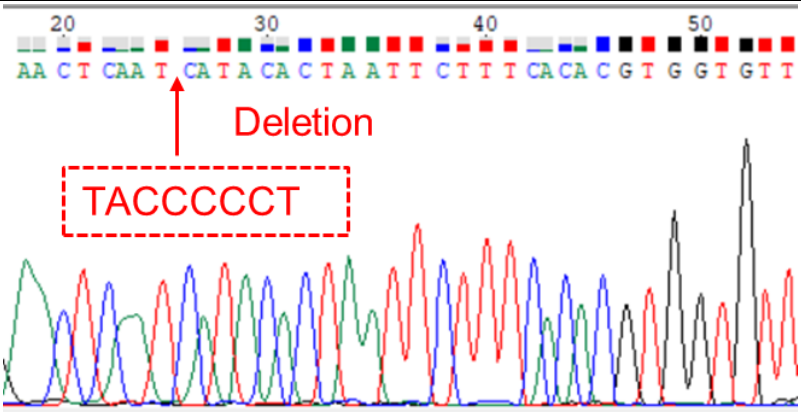
</td></tr>
<tr><td>Sample 43</td><td>AGACTCAAT--------CATACACTAATTCTTTCACACGTGGTGTTT
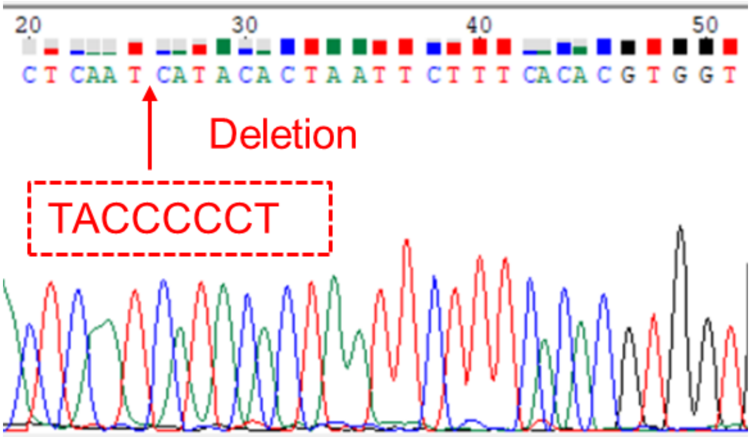
</td></tr>
<tr><td>Sample 55</td><td>AGAACTCAAT--------CATACACTAATTCTTTCACACGTGGTGTTT
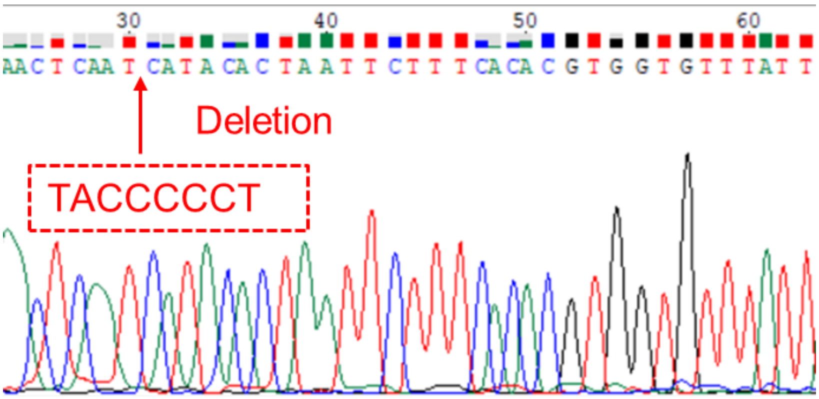

TGGTATAGATCGTTTAGGAAGTCTAA</td></tr>
</table>

<table>
<tr><td></td><td>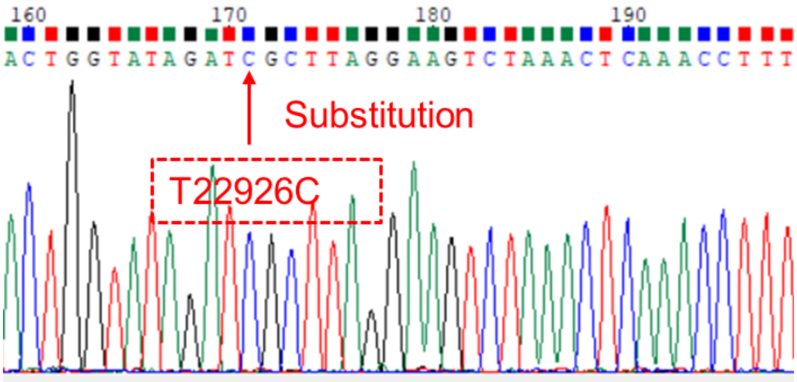
</td></tr>
<tr><td>Sample 56</td><td>AGAACTCAT--------CATACACTAATTCTTTCACACGTGGTGTTT
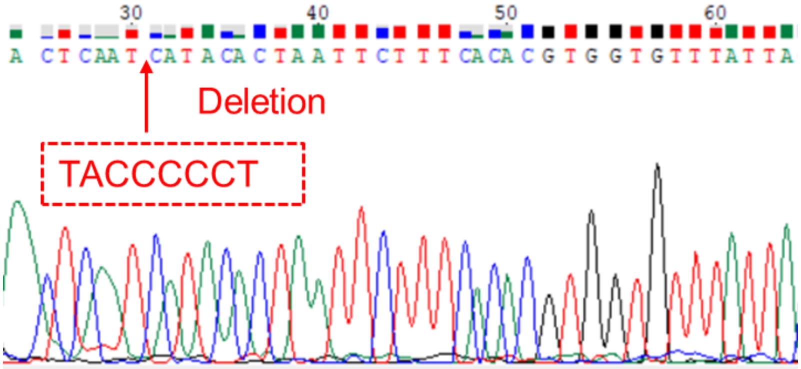

TGGTATAGATCGTTTAGGAAGTCTAA
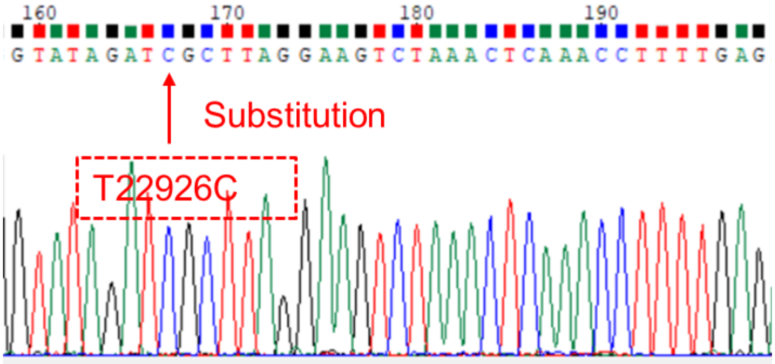
</td></tr>
<tr><td>Sample 58</td><td>AGAACTCAT--------CATACACTAATTCTTTCACACGTGGTGTTT
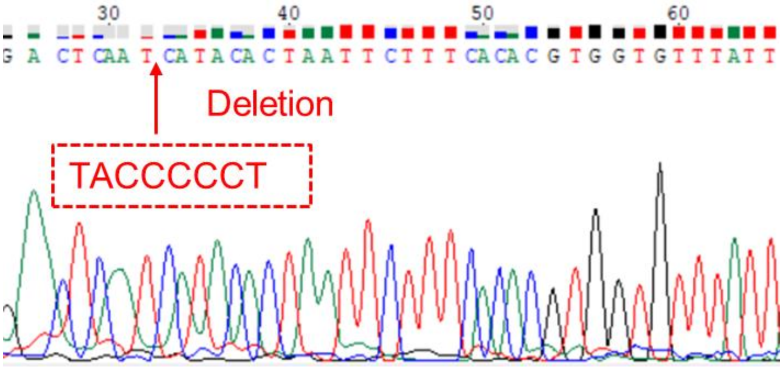

TGGTATAGATCGTTTAGGAAGTCTAA</td></tr>
</table>

	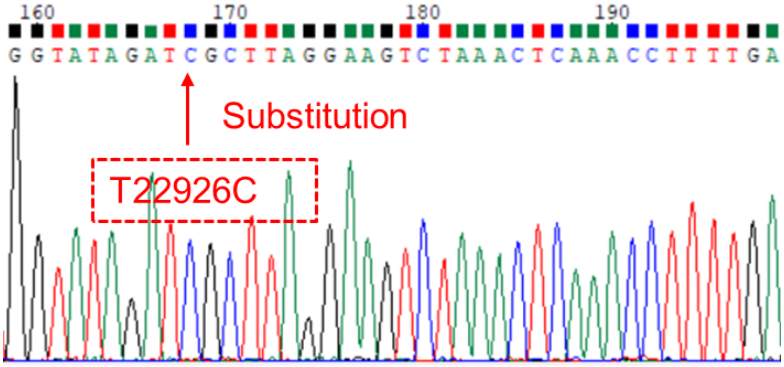
Sample 59	AGAACTCAT--------CATACACTAATTCTTTCACACGTGGTGTTT<br>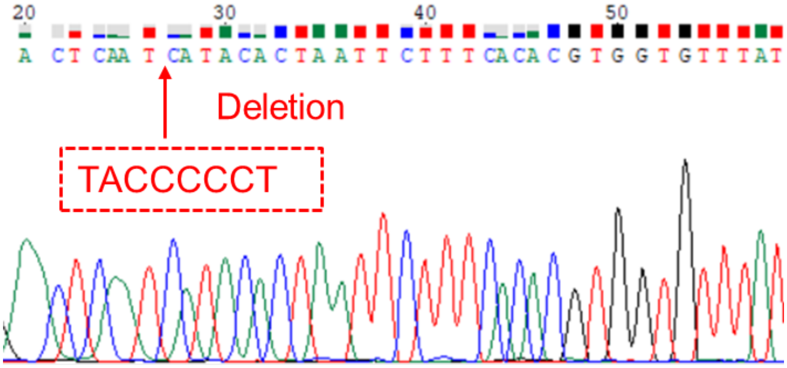 TGGTATAGATCGTTTAGGAAGTCTAA<br>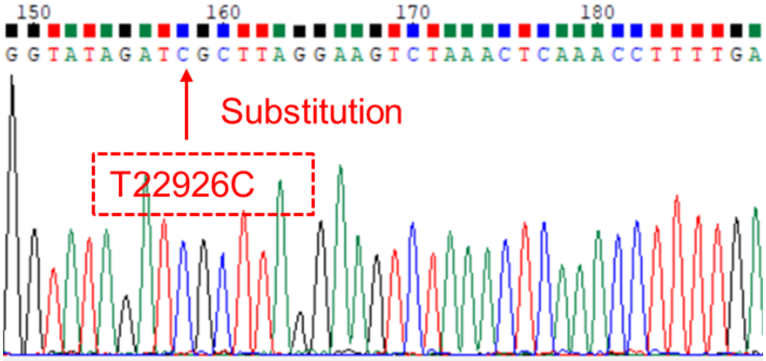
Sample 64	AGAACTCAT--------CATACACTAATTCTTTCACACGTGGTGTTT

	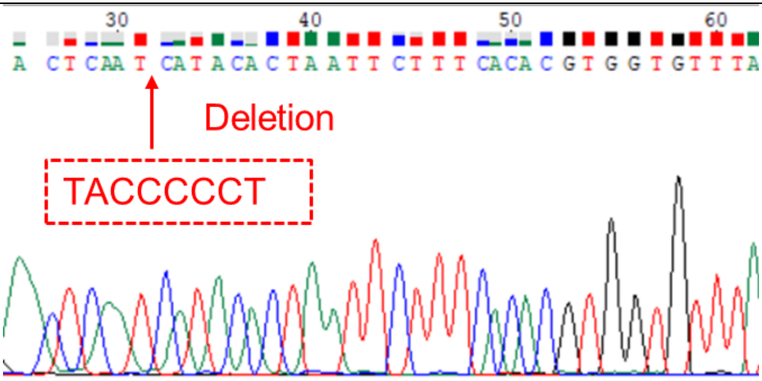 TGGTATAGATCGTTTAGGAAGTCTAA<br>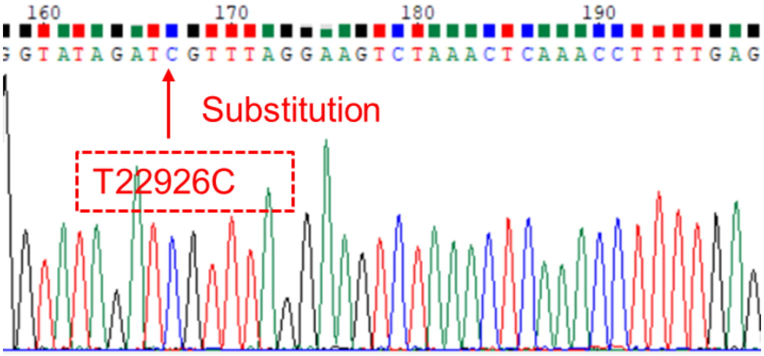
Sample 76	AGACTCAT--------CATACACTAGATTCTTTCCACCTGGTGTTT
	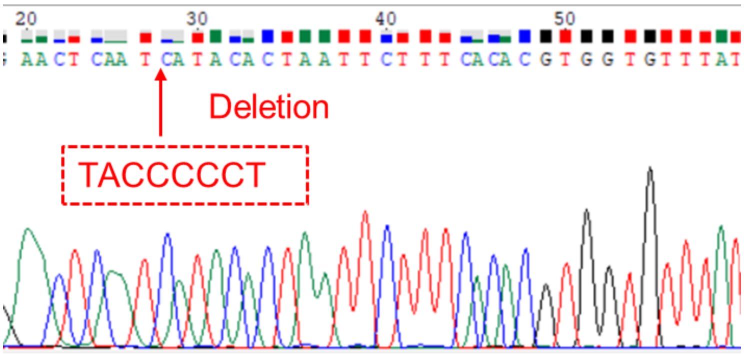 TGGTATAGATCGTTTAGGAAGTCTAA<br>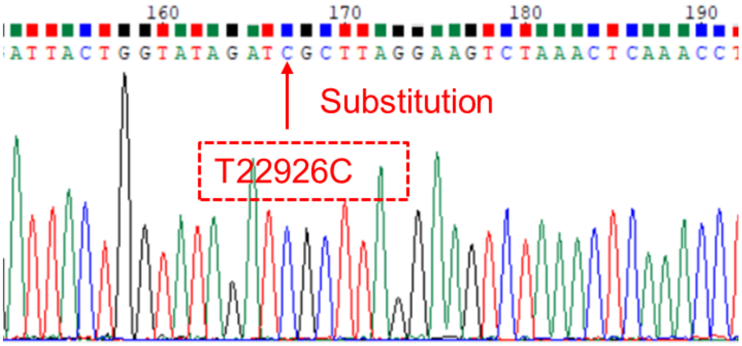
Sample 83	AGAACTCAT--------CATACACTAGATTCTTTCACACGTGGTGTTT

<table>
<tr><td></td><td>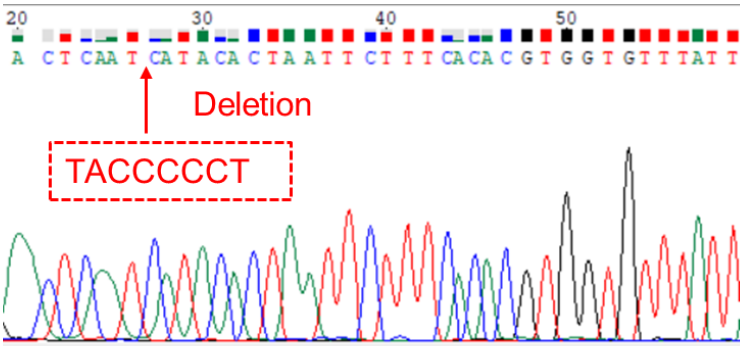
TGGTATAGATCGTTTAGGAAGTCTAA
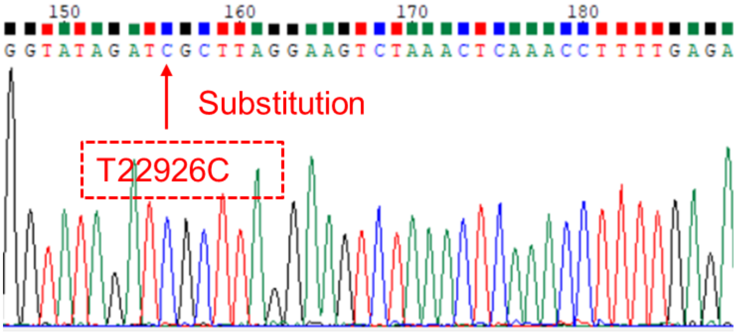</td></tr>
<tr><td>Sample 94</td><td>AGAACTCAT--------CATACACTAATTCTTTCCACGTGGTGTTT
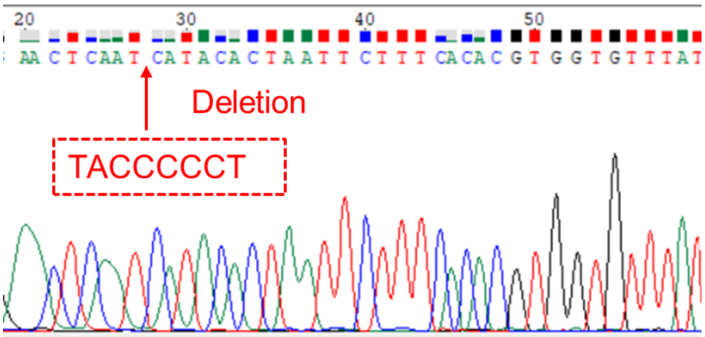
TGGTATAGATCGTTTAGGAAGTCTAA
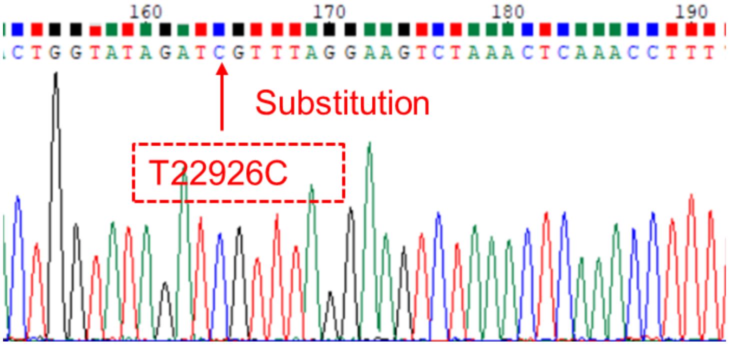</td></tr>
<tr><td>Sample 97</td><td>AGAACTCAT--------CATACACTAATTCTTTCACACGTGGTGTTT</td></tr>
</table>

<table>
<tr>
<td></td>
<td>
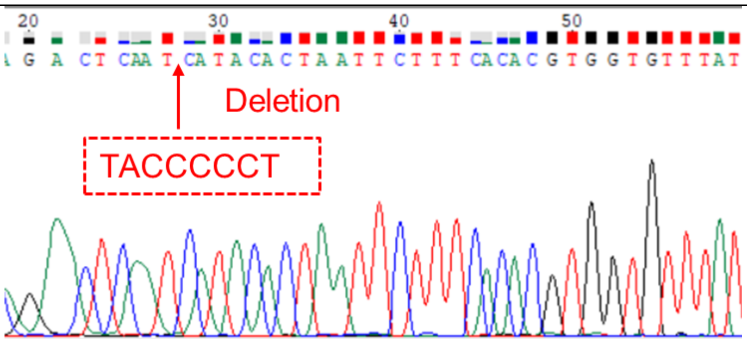

TGGTATAGATCGTTTAGGAAGTCTAA
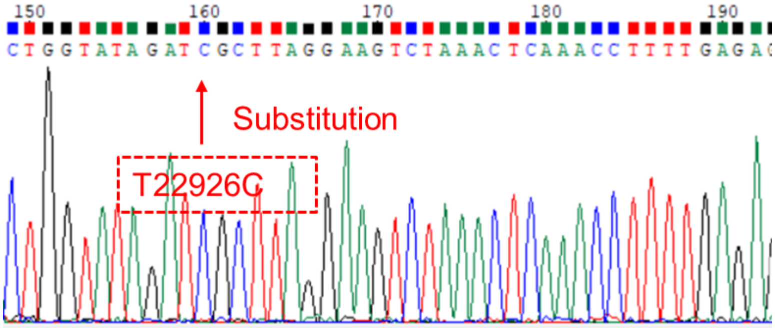

</td>
</tr>
<tr>
<td>Sample 101</td>
<td>
AGACTCAA--------CATACACTAATTCTTTCACACGTGGTGTTT
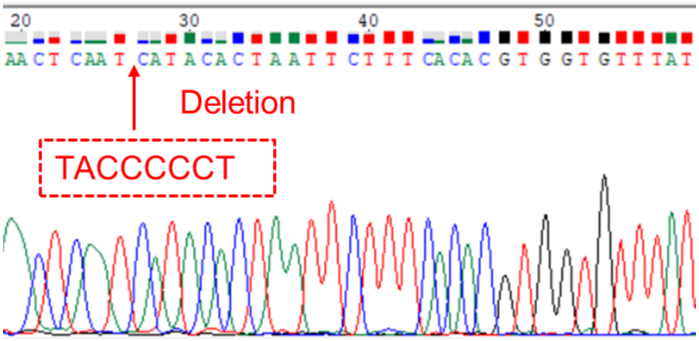

TGGTATAGATCGTTTAGGAAGTCTAA
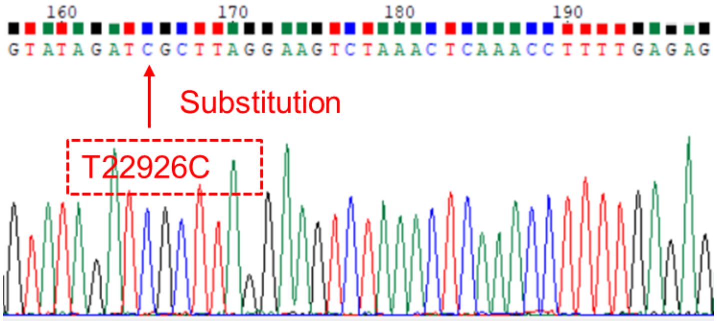

</td>
</tr>
</table>

Table S2. The cycle quantification (Cq) values of the 102 samples by the commercial RT-qPCR targeting ORF1ab, N, and POP7 genes.

Gene / Sample	N (FAM)	ORF 1ab (VIC)	POP7 (CY5)
1	Undetermined	Undetermined	35.17
2	Undetermined	Undetermined	35.77
3	33.31	33.82	34.01
4	Undetermined	Undetermined	34.85
5	33.19	33.34	34.21
6	33.1	33.56	33.76
7	33.11	33.62	34.15
8	33.34	33.65	33.91
9	Undetermined	Undetermined	34.26
10	28.42	29.75	29.96
11	26.03	27.21	35.52
12	28.38	29.73	29.86
13	Undetermined	Undetermined	34.57
14	26.14	27.52	36.69
15	Undetermined	Undetermined	34.2
16	Undetermined	Undetermined	35.05
17	Undetermined	Undetermined	33.18
18	Undetermined	Undetermined	30.36
19	26.11	27.33	35.8
20	Undetermined	Undetermined	33.26
21	Undetermined	Undetermined	30.42
22	Undetermined	Undetermined	30.32
23	Undetermined	Undetermined	30.42
24	Undetermined	Undetermined	30.54
25	Undetermined	Undetermined	32.28
26	21.32	23.57	32.6
27	Undetermined	Undetermined	32.31
28	21.14	23.41	32.26
29	21.01	23.4	32.12
30	28.67	29.99	33.52
31	28.54	29.76	33.21
32	22.51	24.85	29.62
33	29.82	30.6	32.03
34	26.5	27.88	34.45
35	26.76	27.93	33.38

36	22.48	24.72	30.47
37	32.39	34.15	30.99
38	Undetermined	Undetermined	30.72
39	32.37	34.14	31.03
40	21.5	22.45	24.42
41	23.56	25.77	36.22
42	32.48	32.92	32.96
43	33.05	34.26	34.56
44	Undetermined	Undetermined	35.02
45	Undetermined	Undetermined	31.74
46	Undetermined	Undetermined	34.3
47	Undetermined	Undetermined	31.58
48	Undetermined	Undetermined	34.98
49	Undetermined	Undetermined	31.17
50	Undetermined	Undetermined	34.99
51	Undetermined	Undetermined	31.27
52	Undetermined	Undetermined	32.64
53	Undetermined	Undetermined	31.03
54	Undetermined	Undetermined	28.96
55	29.36	30.39	31.13
56	26.59	27.64	34.2
57	Undetermined	Undetermined	28.69
58	25.19	26.13	32.09
59	26.46	27.81	28.13
60	Undetermined	Undetermined	34.93
61	Undetermined	Undetermined	32.54
62	Undetermined	Undetermined	34.96
63	Undetermined	Undetermined	32.42
64	28.65	29.92	33.67
65	Undetermined	Undetermined	31.97
66	Undetermined	Undetermined	31.98
67	Undetermined	Undetermined	31.92
68	Undetermined	Undetermined	33.28
69	Undetermined	Undetermined	30.64
70	Undetermined	Undetermined	33.79
71	Undetermined	Undetermined	30.63
72	Undetermined	Undetermined	30.63
73	Undetermined	Undetermined	30.39
74	Undetermined	Undetermined	30.81

75	Undetermined	Undetermined	30.38
76	21.15	23.38	32.17
77	Undetermined	Undetermined	35.79
78	Undetermined	Undetermined	36.2
79	Undetermined	Undetermined	23.99
80	Undetermined	Undetermined	24.2
81	Undetermined	Undetermined	31.14
82	Undetermined	Undetermined	31.05
83	25.96	27.15	35.65
84	Undetermined	Undetermined	30.22
85	Undetermined	Undetermined	30.35
86	Undetermined	Undetermined	28.9
87	Undetermined	Undetermined	28.91
88	Undetermined	Undetermined	32.07
89	Undetermined	Undetermined	32.08
90	Undetermined	Undetermined	32.77
91	Undetermined	Undetermined	32.05
92	Undetermined	Undetermined	33.11
93	Undetermined	Undetermined	32.93
94	28.32	29.43	29.87
95	Undetermined	Undetermined	32.11
96	Undetermined	Undetermined	32.13
97	32.8	33.14	33.44
98	Undetermined	Undetermined	31.88
99	Undetermined	Undetermined	31.75
100	Undetermined	Undetermined	34.14
101	26.26	27.49	33.93
102	Undetermined	Undetermined	32.07

Table S3. Comparison of isothermal amplification strategies for the detection of SARS-CoV-2 nucleic acids

Strategy	Indepe ndency	Tar get	Dete ction	One-tube	N o.	Reacti on	Tota l	Visu al	Dual visuali	Capa bility	Cost of	Re f.

	from equipment	gene	sensitivity	detection	test steps	temperature and time	detection time	detection capability	zation	for variants surveillance	detection	
RT-LAMP	High	*orf1ab* & *S*	20-200 copies/rxn	Yes	1	63°C for 60 min	60 min	Yes	No	No	Low	[1]
RT-RPA & LAMP	High	*N*	5 virions/rxn	Yes	2	38°C for 15-20 min and then 63°C for 40 min	55-60 min	Yes	No	No	Low	[2]
RT-RPA with an exo probe	Moderate	*N*	7.7 copies/rxn	Yes	1	42°C for 15-20 min	15-20 min	No	No	No	Moderate	[3]
CPA & LFA	High	*N*	200 copies/mL	Yes	2	60-65°C for 60 min	60 min	Yes	No	No	Moderate	[4]
AIOD-CRISPR	High	*N*	~5 copies/rxn	Yes	1	37°C for 40 min	40 min	Yes	No	No	Moderate	[5]
miSHERLOCK	High	*S*	1200 copies/rxn	Yes	1	37°C for 60-120 min	60-120 min	Yes	No	Yes	High	[6]
CRISPR-based DETECTR	High	*N* & *E*	10 copies/rxn	No	2	62°C for 20-30 min and then 37°C for 10 min	30-40 min	Yes	No	No	Moderate	[7]
RT-LAMP &	High	*N* & *E*	150-225 copie	Yes	2	62°C for 30 min	40 min	Yes	No	No	Moderate	[8]

CRISPR/Cas12a			s/rxn			and then 25°C for 10 min						
DAMPR	High	*ORF1, N, and S*	9-13 copies/rxn	No	3	65°C for 30 min, 25°C for 10 min, and finally 25°C for 10 min	50 min	Yes	No	Yes	Moderate	[9]
FEMMAN	Moderate	*S*	1 copy/rxn	Not applicable	3	95°C for 2 min and then 40°C for 30 min	32 min	Yes	No	Yes	High	[10]
**REP-TMAP**	**High**	***S***	**10-200 copies/rxn**	**Yes**	**2**	**42°C for 20 min and then 60°C for 20 min**	**40 min**	**Yes**	**Yes**	**Yes**	**Low**	**This work**

Table S4. The estimated cost of the REP-TMAP assay

	**Volume per reaction**	**Price per kit**	**Volume per kit**	**Cost per reaction**
RNase H2	1 µL	$ 275	25 µL	$ 0.11
REP probe	0.6 µL	$ 181	100 µL	$ 0.216

Revertase	0.1 μL	$ 490	50 μL	$ 0.196
RPA kit	2 μL	$ 585	2832 μL	$ 0.244

Total： $ 0.766

Table S5. The list of all used plasmids in this study

Name	Sequence (5′→3′)	Description
BA2	AGAGAAAACAACAGAGTTGTTATTTCTAGTGATGTTCTTGTTAACAACTAAACGAACAATGTTTGTTTTTCTTGTTTTATTGCCACTAGTCTCTAGTCAGTGTGTTAATCTTATAACCAGAACTCAATCATACACTAATTCTTTCACACGTGGTGTTTATTACCCTGACAAAGTTTTCAGATCCTCAGTTTTACATTCAACTCAGGACTTGTTCTTACCTTTCTTTTCCAATGTTACTTGGTTCCATGCTATACATGTCTCTGGGACCAATGGTACTAAGAGGTTTGATAACCCTGTCCTACCATTTAATGATGGTGTTTATTTTGCTTCCACTGAGAAGTCTAACATAATAAGAGGCTGGATTTTTGGTACTACTTTAGATTCGAAGACCCAGTCCCTACTTATTGTTAATAACGCTACTAATGTTGTTATTAAAGTCTGTGAATTTCAATTTTGTAATGATCCATTTTTGGATGTTTATTACCACAAAAACAACAAAAGTTGGATGGAAAGTGAGTTCAGAGTTTATTCTAGTGCGAATAATTGCACTTTTGAATATGTCTCTCAGCCTTTTCTTATGGACCTTGAAGGAAAACAGGGTAATTTCAAAAATCTTAGGGAATTTGTGTTTAAGAATATTGATGGTTATTTTAAAATATATTCTAAGCACACGCCTATTAATTTAGGGCGTGATCTCCCTCAGGGTTTTTCGGCTTTAGAACCATTGGTAGATTTGCCAATAGGTATTAACATCACTAGGTTTCAAACTTTACTTGCTTTACATAGAAGTT	The 533-bp target sequence inserted into the pUCIDT (Am

		p)<br>pl<br>as<br>mi<br>d
D<br>e<br>lt<br>a	AGAGAAAACAACAGAGTTGTTATTTCTAGTGATGTTCTTGTTAACAAC<br>TAAACGAACAATGTTTGTTTTTCTTGTTTTATTGCCACTAGTCTCTAGTC<br>AGTGTGTTAATCTTAGAACCAGAACTCAATTACCCCCTGCATACACTA<br>ATTCTTTCACACGTGGTGTTTATTACCCTGACAAAGTTTTCAGATCCTC<br>AGTTTTACATTCAACTCAGGACTTGTTCTTACCTTTCTTTTCCAATGTTA<br>CTTGGTTCCATGCTATACATGTCTCTGGGACCAATGGTACTAAGAGGTT<br>TGATAACCCTGTCCTACCATTTAATGATGGTGTTTATTTTGCTTCCATT<br>GAGAAGTCTAACATAATAAGAGGCTGGATTTTTGGTACTACTTTAGAT<br>TCGAAGACCCAGTCCCTACTTATTGTTAATAACGCTACTAATGTTGTTA<br>TTAAAGTCTGTGAATTTCAATTTTGTAATGATCCATTTTTGGATGTTTA<br>TTACCACAAAAACAACAAAAGTTGGATGGAAAGTGGAGTTTATTCTAG<br>TGCGAATAATTGCACTTTTGAATATGTCTCTCAGCCTTTTCTTATGGAC<br>CTTGAAGGAAAACAGGGTAATTTCAAAAATCTTAGGGAATTTGTGTTT<br>AAGAATATTGATGGTTATTTTAAAATATATTCTAAGCACACGCCTATTA<br>ATTTAGTGCGTGATCTCCCTCAGGGTTTTTCGGCTTTAGAACCATTGGT<br>AGATTTGCCAATAGGTATTAACATCACTAGGTTTCAAACTTTACTTGCT<br>TTACATAGAAGTT	T<br>he<br>57<br>5-<br>bp<br>tar<br>ge<br>t<br>se<br>qu<br>en<br>ce<br>in<br>se<br>rte<br>d<br>int<br>o<br>th<br>e<br>p<br>U<br>CI<br>D<br>T<br>(A<br>m<br>p)<br>pl<br>as<br>mi<br>d
B<br>A<br>4	ACAAACTTGTGCCCTTTTGATGAAGTTTTTAACGCCACCAGATTTGCAT<br>CTGTTTATGCTTGGAACAGGAAGAGAATCAGCAACTGTGTTGCTGATT<br>ATTCTGTCCTATATAATTTCGCACCATTTTTCGCTTTTAAGTGTTATGGA	T<br>he<br>58

/5	GTGTCTCCTACTAAATTAAATGATCTCTGCTTTACTAATGTCTATGCAGATTCATTTGTAATTAGAGGTAATGAAGTCAGCCAAATCGCTCCAGGGCAAACTGGAAATATTGCTGATTATAATTATAAATTACCAGATGATTTTACAGGCTGCGTTATAGCTTGGAATTCTAACAAGCTTGATTCTAAGGTTGGTGGTAATTATAATTACCGGTATAGATTGTTTAGGAAGTCTAATCTCAAACCTTTTGAGAGAGATATTTCAACTGAAATCTATCAGGCCGGTAACAAACCTTGTAATGGTGTTGCAGGTGTTAATTGTTACTTTCCTTTACAATCATATGGTTTCCGACCCACTATTGGTGTTGGTCACCAACCATACAGAGTAGTAGTACTTTCTTTTGAACTTCTACATGCACCAGCAACTGTTTGTGGACCTAAAAAGTCTACTAATTTGGTTAAAAACAAATGTGTCAATTTCAACTTCAATGGTTTAACAGGCACAGGTGTTCTTACTGAGTCTAACAAAAAGTTTCTGCCTTTCCAACAATTTGGCAGAGACATTGCTGACACTACTGATGCTGTCCGTGATCCACAGACACTTGAGATTCTTGACATTACACCATGTTCTTTTGGTGGTGTCAGTGTTATAACACCA	2-bp target sequence inserted into the pUCIDT (Amp) plasmid
JN.1	TGTCTCCTACTAAATTAAATGATCTCTGCTTTACTAATGTCTATGCAGATTCATTTGTAATTAAAGGTAATGAAGTCAGCCAAATCGCTCCAGGGCAAACTGGAAATATTGCTGATTATAATTATAAATTACCAGATGATTTTACAGGCTGCGTTATAGCTTGGAATTCTAACAAGCTTGATTCTAAGCATAGTGGTAATTATGATTACTGGTATAGATCGTTTAGGAAGTCTAAACTCAAACCTTTTGAGAGAGATATTTCAACTGAAATCTATCAGGCCGGTAACAAACCTTGTAAAGGTAAAGGTCCTAATTGTTACTTTCCTTTACAATCATATGGTTTCCGACCCACTTATGGTGTTGGTCACCAACCATACAGAGTAGTAGTACTTTCTTT	The 397-bp target sequen

		ce inserted into the pUCIDT (Amp) plasmid
WT-S (downpart)	ATGATCTCTGCTTTACTAATGTCTATGCAGATTCATTTGTAATTAGAGG<br>TGATGAAGTCAGACAAATCGCTCCAGGGCAAACTGGAAAGATTGCTG<br>ATTATAATTATAAATTACCAGATGATTTTACAGGCTGCGTTATAGCTTG<br>GAATTCTAACAATCTTGATTCTAAGGTTGGTGGTAATTATAATTACCTG<br>TATAGATTGTTTAGGAAGTCTAATCTCAAACCTTTTGAGAGAGATATTT<br>CAACTGAAATCTATCAGGCCGGTAGCACACCTTGTAATGGTGTTGAAG<br>GTTTTAATTGTTACTTTCCTTTACAATCATATGGTTTCCAACCCACTAAT<br>GGTGTTGGTTACCAACCATACAGAGTAGTAGTACTTTCTTTTGAACTTC<br>TACATGCACCAGCAACTGTTTGTGGACCTAAAAAGTCTACTA	The 432-bp target sequence inserted into th

		e pUCIDT (Amp) plasmid
WT-S (NTD part)	AGAGAAAACAACAGAGTTGTTATTTCTAGTGATGTTCTTGTTAACAACTAAACGAACAATGTTTGTTTTTCTTGTTTTATTGCCACTAGTCTCTAGTCAGTGTGTTAATCTTACAACCAGAACTCAATTACCCCCTGCATACACTAATTCTTTCACACGTGGTGTTTATTACCCTGACAAAGTTTTCAGATCCTCAGTTTTACATTCAACTCAGGACTTGTTCTTACCTTTCTTTTCCAATGTTACTTGGTTCCATGCTATACATGTCTCTGGGACCAATGGTACTAAGAGGTTTGATAACCCTGTCCTACCATTTAATGATGGTGTTTATTTTGCTTCCACTGAGAAGTCTAACATAATAAGAGGCTGGATTTTTGGTACTACTTTAGATTCGAAGACCCAGTCCCTACTTATTGTTAATAACGCTACTAATGTTGTTATTAAAGTCTGTGAATTTCAATTTTGTAATGATCCATTTTTGGGTGTTTATTACCACAAAAACAACAAAAGTTGGATGGAAAGTGAGTTCAGAGTTTATTCTAGTGCGAATAATTGCACTTTTGAATATGTCTCTCAGCCTTTTCTTATGGACCTTGAAGGAAAACAGGGTAATTTCAAAAATCTTAGGGAATTTGTGTTTAAGAATATTGATGGTTATTTTAAAATATATTCTAAGCACACGCCTATTAATTTAGTGCGTGATCTCCCTCAGGGTTTTTCGGCTTTAGAACCATTGGTAGATTTGCCAATAGGTATTAACATCACTAGGTTTCAAACTTTACTTGCTTTACATAGAAGTT	The 800-bp target sequence inserted into the pUCIDT (Am

		p) pl as mi d

Table S6. The list of all used primers and probes in this study

Name	Sequence (5′→3′)	Description
F3-BA.2	TGTTTTATTGCCACTAGTCTCT	LAMP forward outer primer
B3-BA.2	ATCAAACCTCTTAGTACCATTG	LAMP backward outer primer
FIP-BA.2	CTGAAAACTTTGTCAGGGTAATAAACAGTCA GTGTGTTAATCTTATAACCAG	LAMP forward inner primer
BIP-BA.2	ATCCTCAGTTTTACATTCAACTCAGTGTATAG CATGGAACCAAGT	LAMP backward inner primer
LF-BA.2 for REP-LAMP	TGAAAGAATTAGTGTATG	LAMP forward loop primer
LF-BA.2 for LP-LAMP	TGAAAGAATTAGTGTATGATTGAG	LAMP forward loop primer
LB-BA.2	ACTTGTTCTTACCTTTCTTTTCCAATGT	LAMP backward loop primer
REP-BA.2	CCACGTGTGAAAGAATTAGTGTA/i6FAMdT/G/ rA/TTGAG/3`BHQ1/	Labelled REP for LAMP
LP-BA.2	TGAAAGAATTAGTGTA/i6FAMdT/G/rA/TTGAG 3`BHQ1	Labelled LP for LAMP
CBP-BA.2	/5`6- FAM/TGAAAGAATTAGTGTAG/rA/TTGAGTTC TTTCA/3`BHQ1/	Labelled CBP for LAMP
F3-Delta	GAGGCTGGATTTTTGGTA	LAMP forward outer primer
B3-Delta	GAAAAGGCTGAGAGACAT	LAMP backward outer primer
FIP-Delta	GACTTTAATAACAACATTAGTAGCGCTACTTT AGATTCGAAGACC	LAMP forward inner primer

BIP-Delta	GATCCATTTTTGGATGTTTATTACCGCAATTA TTCGCACTAGAAT	LAMP backward inner primer
LF-Delta	TTATTAACAATAAGTAGGGACTG	LAMP forward loop primer
LB-Delta	CAAAAGTTGGATGGAAAGTG	LAMP backward loop primer
REP-Delta	AACAACAAAAGTTGGATGGAAAG/i6FAMdT/G /rG/AGTTT/3`BHQ1/	Labelled REP for LAMP
F3-BA4/5	GAAATCTATCAGGCCGGTAA	LAMP forward outer primer
B3-BA4/5	GACTTTTTAGGTCCACAAACA	LAMP backward outer primer
FIP-BA4/5	AATAGTGGGTCGGAAACCATATCAAACCTTG TAATGGTGTTG	LAMP forward inner primer
BIP-BA4/5	TTGGTCACCAACCATACAGAGTAGTTGCTGG TGCATGTAGA	LAMP backward inner primer
LF-BA4/5	TAAAGGAAAGTAACAATTAA	LAMP forward loop primer
LB-BA4/5	GTAGTACTTTCTTTTGAACTTCTA	LAMP backward loop primer
REP-BA4/5	GATTGTAAAGGAAAGTAACAAT/i6FAMdT/AA /rC/ACCTG/3`BHQ1/	Labelled REP for LAMP
F3-JN.1	AATTCTAACAAGCTTGATTCTAA	LAMP forward outer primer
B3-JN.1	GTGACCAACACCATAAGT	LAMP backward outer primer
FIP-JN.1	ATAGATTTCAGTTGAAATATCTCTCTCAAGTG GTAATTATGATTACTGGT	LAMP forward inner primer
BIP-JN.1	GGCCGGTAACAAACCTTGGAAACCATATGAT TGTAAAGGA	LAMP backward inner primer
LF-JN.1	GTTTGAGTTTAGACTTCCTA	LAMP forward loop primer
LB-JN.1	TAAAGGTAAAGGTCCTAATTGTTACTT	LAMP backward loop primer
REP-JN.1	AAAGGTTTGAGTTTAGACTTCCT/i6FAMdT/AA AC/rG/ATCTA/3`BHQ1/	Labelled REP for LAMP
F3-POP7	GGTGGCTGCCAATACCTC	LAMP forward outer primer

B3-POP7	ACTCAGCATGCGAAGAGC	LAMP backward outer primer
FIP-POP7	GTTGCGGATCCGAGTCAGTGGCCGTGGAGCT TGTTGATGA	LAMP forward inner primer
BIP-POP7	AACTCAGCCATCCACATCCGAGTCACGGAGG GGATAAGTGG	LAMP backward inner primer
LF-POP7	TGTCGGTCTCTGGCTCCAGC	LAMP forward loop primer
LB-POP7	AGGGTCACACCCAAGTAATT	LAMP backward loop primer
REP-POP7	TCTTCAGGGTCACACCCAAGTAA/i6FAMdT/T/r G/AAAAG/3`BHQ1/	Labelled REP for LAMP
F-T7-BA2	TAATACGACTCACTATAGGGGTTAACAACTA AACGAACAA	PCR forward primer with T7 promotor for RNA preparation
R-T7-BA2	TGATGTTAATACCTATTGGC	PCR backward primer for RNA preparation
F-T7-Delta	TAATACGACTCACTATAGGGGTTAACAACTA AACGAACAA	PCR forward primer with T7 promotor for RNA preparation
R-T7-Delta	TGATGTTAATACCTATTGGC	PCR backward primer for RNA preparation
F-T7-BA4/5	TAATACGACTCACTATAGGGATGATCTCTGC TTTACTAATGT	PCR forward primer with T7 promotor for RNA preparation
R-T7-BA4/5	AGTAGACTTTTTAGGTCCACA	PCR backward primer for RNA preparation
F-T7-JN1	TAATACGACTCACTATAGGGGTCTCCTACTA AATTAAATGATCT	PCR forward primer with T7 promotor for RNA preparation

R-T7-JN1	TACTACTCTGTATGGTTGGTG	PCR backward primer for RNA preparation
F-T7-POP7	TAATACGACTCACTATAGGGAACCGCGCCAT CAACATCG	PCR forward primer with T7 promotor for RNA preparation
R-T7-POP7	CACACTCAGGAAGGCCCACT	PCR backward primer for RNA preparation
FP-MT-up	TGTTTTATTGCCACTAGTCTCT	Forward primer for RT-PCR plus sequencing
RP-MT-up	TAAGAAAAGGCTGAGAGACAT	Backward primer for RT-PCR plus sequencing
FP-MT-down	TGATCTCTGCTTTACTAATGTCTAT	Forward primer for RT-PCR plus sequencing
RP-MT-down	AGGTCCACAAACAGTTGC	Backward primer for RT-PCR plus sequencing

References

[1] C. Yan, J. Cui, L. Huang, B. Du, L. Chen, G. Xue, S. Li, W. Zhang, L. Zhao, Y. Sun, *Clin. Microbiol. Infect.* **2020**, *26* (6), 773.

[2] J. Song, M. El-Tholoth, Y. Li, J. Graham-Wooten, Y. Liang, J. Li, W. Li, S. R. Weiss, R. G. Collman, H. H. Bau, *Anal. Chem.* **2021**, *93* (38), 13063.

[3] O. Behrmann, I. Bachmann, M. Spiegel, M. Schramm, A. Abd El Wahed, G. Dobler, G. Dame, F. T. Hufert, *Clin. Chem.* **2020**, *66* (8), 1047.

[4] a) S. Wu, X. Shi, Q. Chen, Y. Jiang, L. Zuo, L. Wang, M. Jiang, Y. Lin, S. Fang, B. Peng, *Ann. Clin. Microbiol. Antimicrob.* **2021**, *20* (1), 38; b) USTAR, *The novel coronavirus (2019-nCoV) real-time molecular diagnostic system* **2022**, *https://www.bioustar.com/intro/19.html*.

[5] X. Ding, K. Yin, Z. Li, R. V. Lalla, E. Ballesteros, M. M. Sfeir, C. Liu, *Nat. Commun.* **2020**, *11* (1), 4711.

[6] H. De Puig, R. A. Lee, D. Najjar, X. Tan, L. R. Soenksen, N. M. Angenent-Mari, N. M. Donghia, N. E. Weckman, A. Ory, C. F. Ng, *Sci. Adv.* **2021**, *7* (32), eabh2944.

[7] J. P. Broughton, X. Deng, G. Yu, C. L. Fasching, V. Servellita, J. Singh, X. Miao, J. A. Streithorst, A. Granados, A. Sotomayor-Gonzalez, *Nat. Biotechnol.* **2020**, *38* (7), 870.
[8] B. Pang, J. Xu, Y. Liu, H. Peng, W. Feng, Y. Cao, J. Wu, H. Xiao, K. Pabbaraju, G. Tipples, *Anal. Chem.* **2020**, *92* (24), 16204.
[9] J. Song, B. Cha, J. Moon, H. Jang, S. Kim, J. Jang, D. Yong, H.-J. Kwon, I.-C. Lee, E.-K. Lim, *ACS Nano* **2022**, *16* (7), 11300.
[10] Y. Liu, Y. Yang, G. Wang, D. Wang, P.-L. Shao, J. Tang, T. He, J. Zheng, R. Hu, Y. Liu, *Nat. Biomed. Eng.* **2023**, *7* (12), 1636.
